# Characterization of “Free Base” and Metal Complex Thioalkyl Porphyrazines by Magnetic Circular Dichroism and TDDFT Calculations

**DOI:** 10.1021/acs.jpcb.0c09277

**Published:** 2020-12-22

**Authors:** Simone Ghidinelli, Sergio Abbate, Ernesto Santoro, Sandra Belviso, Giovanna Longhi

**Affiliations:** † Dipartimento di Medicina Molecolare e Traslazionale, Università di Brescia, Viale Europa 11, 25123 Brescia, Italy; ‡ Research Unit of Brescia, CNR, Istituto Nazionale di Ottica (INO), c/o CSMT, via Branze 45, 25123 Brecia, Italy; § Dipartimento di Scienze, Università della Basilicata, Viale dell’Ateneo Lucano 10, 85100 Potenza, Italy

## Abstract

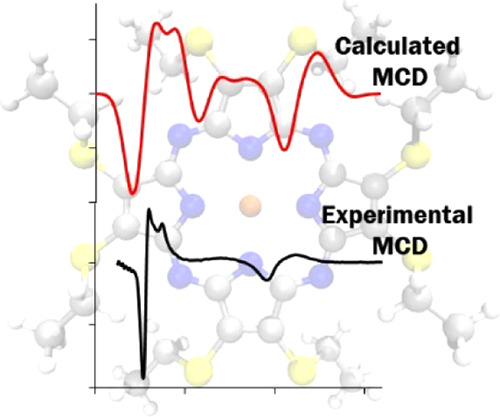

UV–vis absorption and magnetic circular dichroism (MCD) spectra of octakis thioethyl “free base” porphyrazine H_2_OESPz and its metal complexes MOESPz (M = Mg, Zn, Ni, Pd, Cu), as well as of [MnOESPz(SH)] were recorded. In the last case, MCD proved to have quite good sensitivity to the coordination of this complex with 1-methylimidazole (1-mim) in benzene. Time-dependent density functional theory (TDDFT) calculations were carried out for the considered porphyrazine complexes and showed good performance on comparing with MCD and UV–vis experimental spectra, even in the open-shell Cu and Mn cases. Calculations accounted for the red shift observed in the thioalkyl compounds and allowed us to reveal the role of sulfur atoms in spectroscopically relevant molecular orbitals and to highlight the importance of the conformations of the thioethyl external groups. Calculated MCD spectra of [MnOESPz(SH)] confirm the Mn(III) → Mn(II) redox process, which leads to the [Mn(OESPz)(1-mim)_2_] species, and the relevance of the spin state for MCD is revealed.

## Introduction

The highly delocalized π-electron system of tetrapyrrole macrocycles makes these systems ideal substrates for optoelectronics. In fact, porphyrins and phthalocyanines, the two most important subgroups of this family, find applications as dyes in organic photovoltaics (OPV)^1,2^ and for the development of materials for nonlinear optics (NLO).^3−6^ The structurally related porphyrazine macrocycles^7−10^ have, however, been much less studied in this field, although they display interesting structural and optical properties. In fact, they allow ample and facile synthetic modularity, display a wider UV–vis absorption range, promising the development of panchromatic photovoltaic materials and, under some circumstances, can give rise to columnar liquid crystal mesophases.^11−15^ Very few examples of porphyrazine applications in NLO^16−24^ and OPV^25,26^ have been reported, and only very recently some of us have described the potentiality of nonsymmetrically substituted thioalkyl porphyrazines in OPV^27,28^ and NLO.^29,30^ For the development of new optoelectronic materials based on the porphyrazine framework, a detailed knowledge of their electronic structure is of utmost importance. For this purpose, magnetic circular dichroism (MCD)^31−37^ is quite a useful tool, displaying very attractive features with respect to standard UV–vis spectroscopy. In fact, MCD is more sensitive to the molecular electronic properties and, among others, offers the possibility of identifying degenerate excited electronic states because the resulting positive and negative bands make it possible to resolve overlapping electronic transitions. Such spectroscopy, also, often provides key information about the redox and spin state of the central metal. The basis of the MCD theory was developed in the 1960–1970s.^38,39^ A further key development of this technique has been provided by the recent application of density functional theory (DFT) computations for MCD spectra simulation;^40−47^ correspondence between simulated and experimental spectra provides confidence in the picture obtained by simple time-dependent DFT (TDDFT) calculations, however, with due attention to possible TDDFT limitations, particularly in the case of open-shell systems.^48,49^

**Scheme 1:**
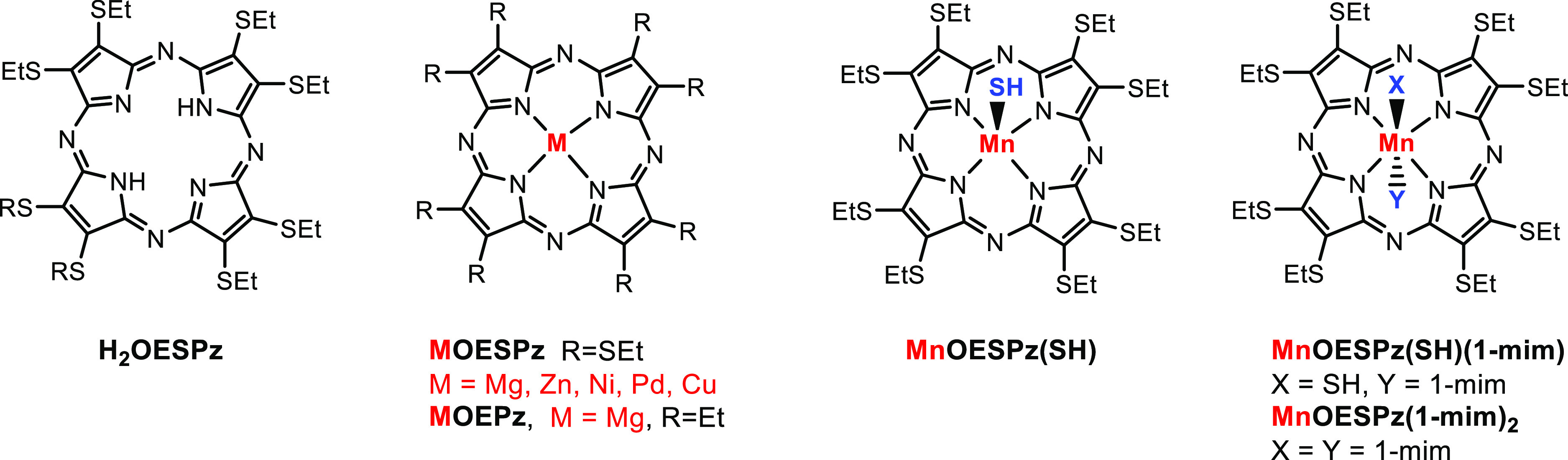
Structures of the Studied Compounds: “Free Base” H_2_OESPz, Octakis Thioethyl MOESPz Complexes, the Octakis Ethyl MgOEPz Complex, and Axially Coordinated Mn(II) and Mn(III) Complexes

The MCD technique has been widely employed not only for investigating porphyrins’ and phthalocyanines’ electronic structures,^32,33,50−53^ but also porphyrazine macrocycles^53,54^ have been investigated; considering thioalkyl porphyrazines,^55−58^ only a study by Stillman et al. is present in the literature^59^ to the best of our knowledge. This prompted us to carry out an extensive MCD characterization on 2,3,7,8,12,13,17,18-octakis(ethylsulfanyl)-5,10,15,20-porphyrazine (OESPz) in its “free base” form H_2_OESPz, its d^0^ Mg(II) complex, and a full series of transition-metal complexes: d^10^ Zn(II) and d^8^ Ni(II) and Pd(II) complexes, open-shell d^9^ Cu(II), and the d^7^/d^6^ Mn(II)/Mn(III) redox couple (see Scheme 1).^56^ TDDFT computations on selected examples offer deep insight into their electronic structure. It is worthwhile to recall that the Mn(II) thioethyl porphyrazine complex has been reported to possess interesting catalytic properties affording easy removal of halogen atoms from halogenated hydrocarbons via oxidative addition to manganese.^55−58^ This allows even defluorination of organic halides, proceeding through activation of the scarcely reactive C–F bond, thus paving the way to environmental applications for dehalogenation of dangerous organic pollutants.^56^ Such a dehalogenation process occurs through an interconversion of the Mn(II)/Mn(III) redox couple, which was investigated through absorption spectroscopy, since the two species have different typical UV–vis spectral features.^56^ The same redox process is investigated here by MCD spectroscopy, which may, in principle, guarantee a higher sensitivity and selectivity than UV–vis absorption analysis. In fact, even if the MCD signal arises from the same transitions as those determining the UV–visible absorption spectrum, the selection rules are different. Moreover, the presence of positive/negative features permits us to better resolve different adjacent almost degenerate transitions such that the correspondence theory–experiment is for sure more stringent.

## Experimental Section

### General Procedures

All chemicals and solvents (Aldrich) were of reagent grade. Solvents were dried and distilled before use according to standard procedures.

### Synthesis

Compounds H_2_OESPz,^11−15,60,61^ MgOESPz,^11,12,62^ ZnOESPz,^11^ NiOESPz,^11−15^ PdOESPz,^28^ CuOESPz,^11−15,62^ and [(MnOESPz)(SH)]^55,56^ were prepared following previously reported procedures. Their spectroscopic data matched those reported here.

### Spectroscopic Measurements

UV–vis and MCD spectra were recorded in the 250–800 nm range using a J-815SE spectrometer, with a home-built cell holder equipped with a 0.6 T permanent magnet, in 1-cm pathlength quartz cells (the concentration of the solutions was ca. 10^–5^–10^–6^ M) at room temperature, 200 nm/min scanning speed, and 10 scans per measurement. For each sample, the two magnetic field orientations were tested and enantiomericity of the two magnetic field directions was checked.

### Computational Methods

Calculations were performed with ADF program 2018.105.^63,64^ The BP86 functional combined with Grimme’s D3 correction^65^ and the TZP basis set was employed for structure optimization. Excitation energies, oscillator strengths, and MCD parameters^40−42,66^ were computed by TDDFT formalism using the M06-L functional and TZP basis set. Molecular orbitals were generated by Molden software.^67^ For open-shell compounds (copper and manganese complexes), the spin-unrestricted formalism was employed using the same functional and basis set used for the closed-shell molecules. Scalar ZORA^68,69^ approximation was used for the relativistic effects. Electronic excitation energies were found with the Davidson procedure for open-shell systems in a spin-unrestricted TDDFT calculation with scalar ZORA and no frozen core.

## Results and Discussion

### Absorption and MCD Spectra

Porphyrazines MOESPz were prepared as already described in refs (11) and (55−58).

**Figure 1:**
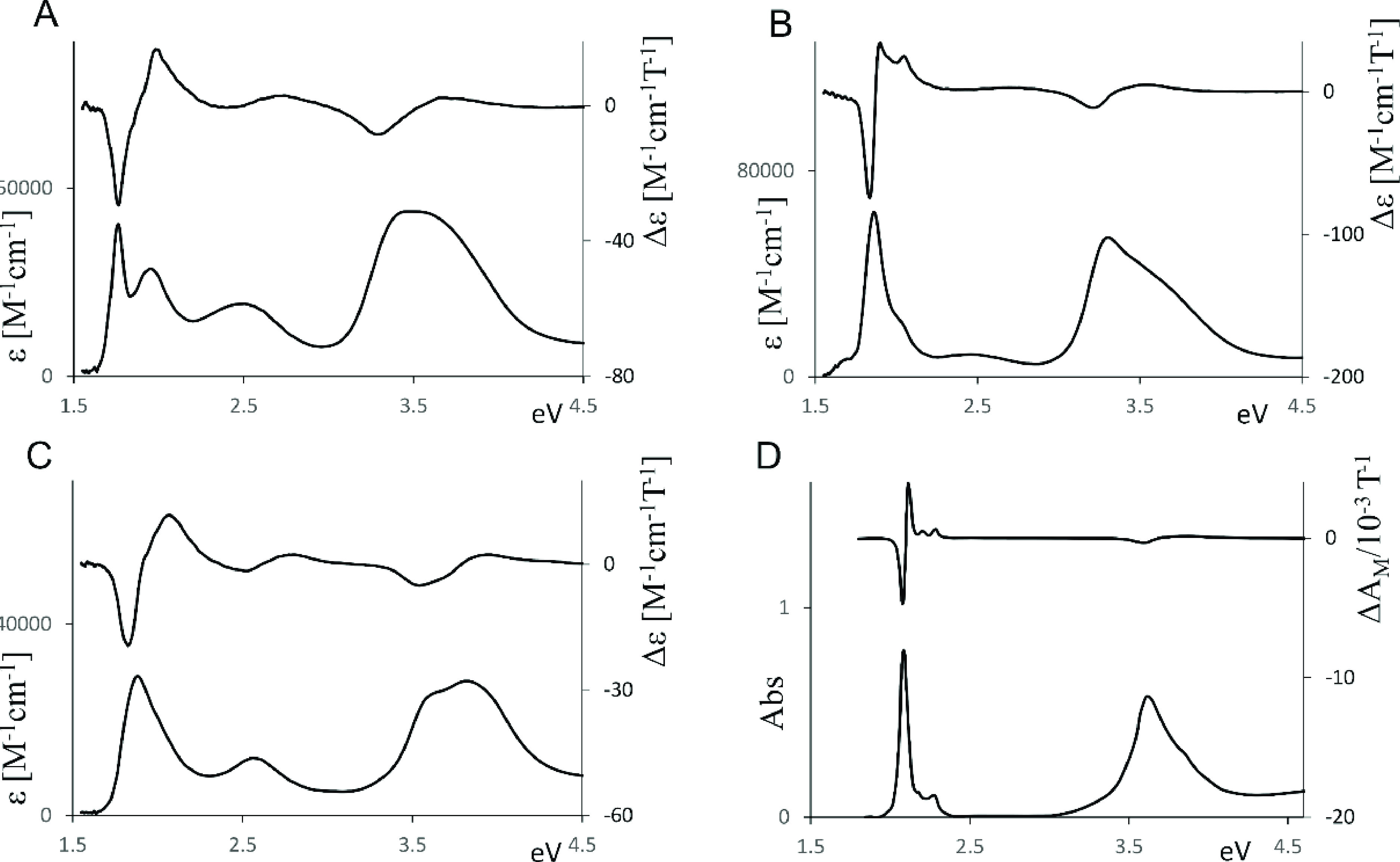
Experimental absorption and MCD spectra of “free base” thioethyl porphyrazine H_2_OESPz (A), MgOESPz (B) and NiOESPz (C) thioethyl porphyrazine complexes, and Mg ethylporphyrazine MgOEPz (D); the last data have been redrawn from ref (59).

UV–vis and MCD spectra have been recorded in dichloromethane (DCM) in the 250–800 nm range. We report in Figure 1, the spectra of H_2_OESPz, NiOESPz, MgOESPz and, for comparison and discussion, absorption and MCD spectra of an analogous alkyl-substituted Mg complex, which is Mg-octaethyltetraazaporphyrin MgOEPz, taken from ref (59).

### “Free Base” H_2_OESPz

All UV–vis spectra exhibit the typical features of nonaggregate thioalkyl porphyrazines; for the “free base”, the two Q*_x_* and Q*_y_* bands can be easily distinguished at 1.77 eV (704 nm) and at 1.94 eV (635 nm), respectively; the Soret band is observed at about 3.53 eV (357 nm), and its shape suggests contributions from two bands at about 3.43 (361 nm) and 3.58 eV (346 nm). Both the Q and B features are red-shifted with respect to alkyl-substituted porphyrazines. Furthermore, as typical of the thioethyl porphyrazine, there is an intermediate broad, less intense, “extra” band between the Q and B features, at about 2.53 eV (495 nm). This feature (in ref (70) called the W band) in early studies had been associated with a n_sulfur_ → π* transition,^29,70,71^ and, more recently, based on calculations, was attributed to a shifted B component^72^ of the Soret band.^73^ The MCD spectra are similar but not identical for all examined compounds as we are going to comment below. Considering H_2_OESPz, only Faraday B-terms are expected, in accordance with the other *D*_2*h*_ symmetry (or lower) metal-free tetrapyrroles.^37,74^ In fact, two B-terms are associated with the Q*_x_* and Q*_y_* transitions, with a negative band at 1.76 eV (705 nm) and a positive one at 2 eV (622 nm). The sign of the Q doublet is related^34,35^ to the energy difference between the first two excited states, which is lower than the energy difference between the two occupied states involved in the dipole allowed transitions (ΔLUMO < ΔHOMO considering Gouterman orbitals^75^). Analogously a (−, +) doublet is observed in correspondence to the UV Soret band with the negative band at 3.3 eV (377 nm) and the positive one at 3.64 eV (339 nm). Such spectral features closely resemble those displayed by tetra-*tert*-butyl porphyrazine.^52^ The MCD spectrum of H_2_OESPz also displays a positive band at 2.7 eV (461 nm), which does not exactly correspond to the UV–vis “extra” band located at a longer wavelength. All assignments will be discussed in the following, based on the results of TDDFT calculations.

### Metal Complexes

Metal complexes of symmetrically substituted porphyrazines present a *D*_4*h*_ symmetry core, if one disregards possible distortions from the planarity of the core itself or the pendant conformational degree of freedom (see the following Results and Discussion); therefore, in their MCD spectra Faraday A-term features are expected in correspondence to the main UV–vis absorption bands.^34−37^ Due to the higher molecular symmetry, the UV–vis spectra of the porphyrazine metal complexes MOESPz display a single Q band, which is blue-shifted (ca. 40–45 nm = 0.10–0.11 eV) compared to that of the corresponding “free base”. In fact, the MCD spectra of the investigated metal complexes present the typical features whose assignment can be based on the Gouterman four-orbital scheme^75^ or the Michl perimeter model^76^ based on the electronic description by Moffitt^77^ and Michl;^34^ these electronic models account for the most intense features observed for all porphyrinoids. Based on these schemes, the lowest unoccupied levels are 2-fold degenerate π* orbitals, thus justifying the presence of A-terms; in the case of porphyrins, the highest energy occupied levels are π orbitals a_2u_ and a_1u_ for *D*_4*h*_ symmetry; these states are nearly degenerate in the case of porphyrin, while are well separated in energy in porphyrazines.^53,78,79^ In fact, in the latter case the presence of electronegative nitrogen atoms in the meso position lowers the energy of a_2u_ states with respect to that of a_1u_ states.^53^ This fact allows mixing of the forbidden Q transitions and allowed B transitions, which results in the intensification of the Q band. In general, calculations indicate that the metal center has only a minor influence on the energy values of both the higher occupied and lower unoccupied orbitals for porphyrazine metal complexes.^53^ MCD spectra of d^0^ Mg(II) and d^10^ Zn(II) complexes appear almost superimposable (see Figure S1 in the Supporting Information (SI)) but also the d^9^ Cu(II) complex shows a similar Q feature. In particular, the MCD spectral shape of the Mg(II) complex (Figure 1B) suggests the presence of an intense Faraday A-term feature centered at about 1.87 eV (664 nm), in correspondence to the UV–vis maximum of the Q band at 1.85 eV (671 nm); however, such a spectral feature is nonsymmetrical, and its positive higher energy branch partly overlaps with other A- and/or B-terms allied to the feature at 2.36 eV (609 nm, precise assignment will be discussed in the following). Finally, a weaker Faraday A-term centered at 3.41 eV (364 nm) is associated with the UV–vis Soret broad feature at 3.81–3.54 eV (325–350 nm).

The Ni(II) and Pd(II) OESPz complexes display very similar UV–vis and MCD spectra (Figure S2 in the SI), which are also similar to those for the “free base”, especially considering the Q region, but are quite different from those of the Mg, Zn, and Cu complexes. The experimental MCD spectrum of the d^8^ Ni complex shows (Figure 1C) an asymmetric positive Faraday A-term centered at 1.91 eV (650 nm), in correspondence to the maximum of the Q band at the same wavelength; the origin of this asymmetry needs calculations for a correct assignment. In correspondence to the broad Soret band located at around 3.54 eV (350 nm), a positive A-term is expected and is indeed observed, again presenting broadness and asymmetry. Finally, a positive MCD feature centered at 2.58 eV (480 nm), approximately in correspondence to the “extra” band absorption at 2.56 eV (484 nm) is observed.

The thioalkyl complexes of this work may be compared to the octaethyl Mg(II) complex in which the sulfur atoms are missing at the periphery.^59^ In the MgOEPz complex the Q band maximum is blue-shifted to 2.03 eV (612 nm), with a second weaker band at 2.21 eV (562 nm) affected by vibronic contributions; the Soret band at 3.48 eV (356 nm) is also blue-shifted, but only slightly. The MCD spectrum exhibits an intense, positive, and almost symmetrical Faraday A-term centered at 2.03 eV (610 nm) in correspondence to the maximum of the Q band, followed by a positive band at 2.21 eV (562 nm). A weak positive A-term is found in correspondence to the maximum of the Soret band. A comparison of experimental MCD data and assignments for MgOESPz and MgOEPz have already been presented and discussed by Stillman,^59^ and for completeness we recall those data to be commented with the aid of TDDFT calculations.

### TDDFT Calculations

To gain insight into the properties of the examined systems we considered DFT optimization, followed by TDDFT calculations, thus obtaining energy levels, characteristics of the allowed transitions, and spectroscopic responses. These results, on one hand, will give solid ground for data interpretation and spectroscopic assignments and, at the same time, the good correspondence between observed and calculated spectra will allow us to validate the calculations.

### Conformational Aspects

Before proceeding with electronic properties, characterization, and discussion, it is worthwhile to mention about pendant groups’ conformational mobility, particularly keeping in mind that sulfur atoms may, and in fact do, contribute to the molecular orbitals involved in the spectroscopically observed transition and may influence the symmetry of the system. In ref (78), the problem for a Ni(II) alkylthioporphyrin and alkylthioporphyrazine was analyzed.^79^ In principle, the macrocycle may deviate from planarity, depending on the pendant group orientation; however, in the cases of porphyrazines, this effect seems negligible, but symmetry considerations have to be taken into account while discussing sulfur orbital contribution to transitions. Our performed calculations confirmed the results of the studies conducted in refs (78) and (79) where the thioalkyl chains lowest energy conformation was such that the four-coordinate metal porphyrins have *D*_4_ symmetry (which reduces to *D*_2_ symmetry for the metal-free compound), with an alternating up and down orientation of the pendant groups (*udud*), while in the case of porphyrazines, the thioalkyl chains manifest the lowest energy conformation with the up-up-down-down orientation (*uudd*), which makes the four-coordinate metal complexes have *D*_2*d*_ symmetry (while the “free base” presents *C*_2*v*_ symmetry). Calculations herein presented refer to this last structure (*uudd*); however, one may well expect many conformers in solution. For this reason, comparison with a different symmetry pattern (*udud*), that is the most commonly studied *D*_*2*_ (free base) and *D*_4_ (four coordinated metal complexes), will be presented below.

### Energy Levels and Orbitals

**Figure 2:**
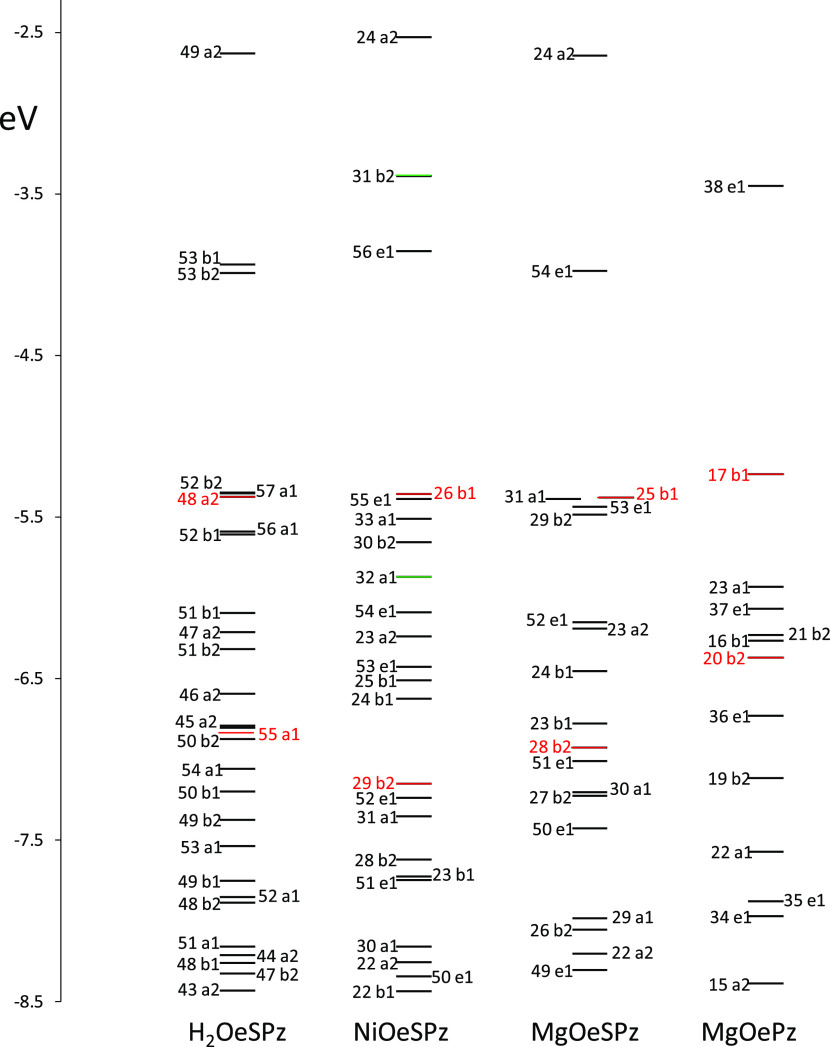
Calculated energy levels of H_2_OESPz, NiOESPz, and MgOESPz complexes, and Mg ethylporphyrazine MgOEPz (occupied Gouterman orbitals written in red).

We report in Figure 2, the calculated energy levels of the four significant cases corresponding to the experimental data presented in Figure 1: the “free base”, Ni metal complex, Mg metal complex, and the alkyl-substituted MgOEPz complex. The relevant molecular orbitals are presented in Figure S3A,B. The lowest unoccupied molecular orbital (LUMO) (corresponding to the Gouterman LUMO orbital) presents similar energies and similar atomic contributions in the three thioalkyl compounds. In the case of the free base, the two orbitals 53b1 and 53b2 are nearly degenerate and similar to 56e and 54e of Ni and Mg complexes, respectively; they are all similar in shape and atomic contribution to the degenerate state 38e1 of the MgOEPz, which, however, is found at a higher energy. Similarly, the first occupied orbitals of the Gouterman type (responsible for the Q bands) are similar in the four cases (48a2, 26b1, 25b1, and 17b1); however, the thiolate compounds show important contributions from sulfur atoms. The energy value is quite similar for the three thiolate compounds, while it is slightly higher in the case of MgOEPz, so that the optical gap corresponding to the first optical transition is expected at a lower energy in the thio-compounds, as in fact observed. The lower energy Gouterman orbitals (55a1, 29b2, 28b2, and 20b2) involved in the B transitions are very similar in the four cases since sulfur atoms are only marginally involved, while a large contribution originates from nitrogen atoms, as expected; since this orbital is localized within the macrocycle, it is similar to that calculated for the MgOEPz complex (see Figure S3B). The corresponding energy level displays some slight differences in the considered complexes; in the presence of nickel, the energy is lower as compared to the metal-free case or the MgOESPz complex; in the case of MgOEPz, one calculates a higher energy but the difference is not as pronounced as that for the highest occupied molecular orbital (HOMO) case. This explains why the blue shift of the Soret band of MgOEPz is lower than what was observed for the Q band. In Figure S3A,B other orbitals involved in optically active transitions are reported and most of them show large contributions from sulfur atoms. Among the metal orbitals, in the energy range of the observed bands, one can recognize the nickel orbitals (occupied 32a1, unoccupied 31b2), while the corresponding ones for Mg are not in the range of interest.

### Calculated Spectra

**Figure 3:**
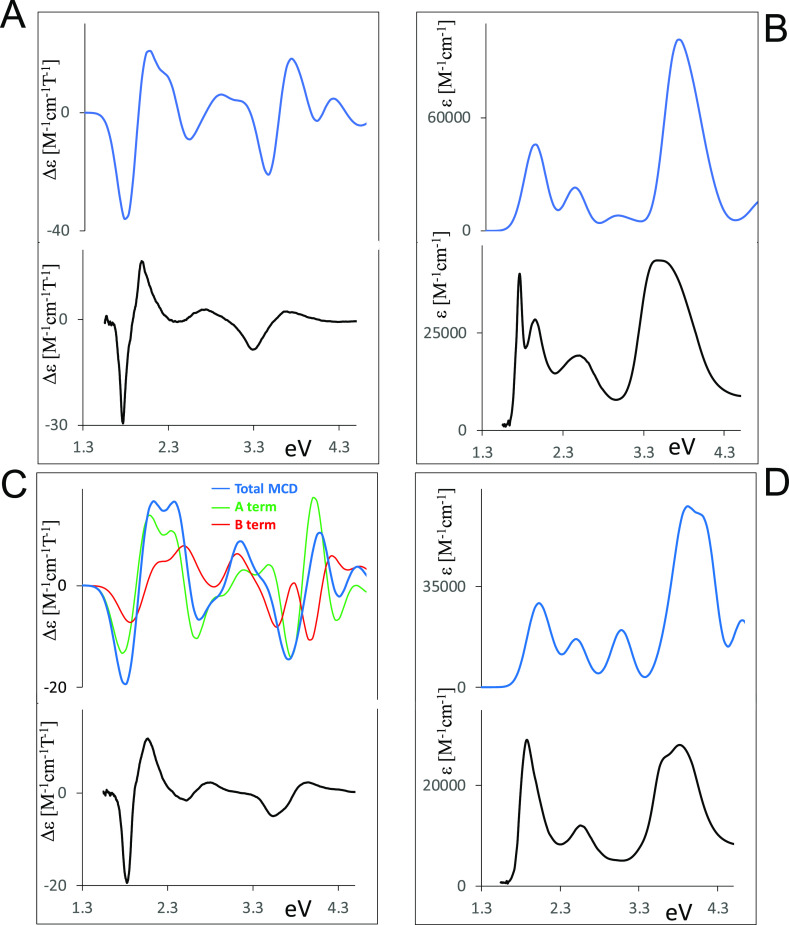
Comparison of experimental (black lines) and calculated (colored lines) UV–vis and MCD spectra. (A) Calculated and experimental MCD spectra of H_2_OESPz. (B) Calculated and experimental UV–vis spectra of H_2_OESPz. (C) Calculated and experimental MCD spectra of NiOESPz. (D) Calculated and experimental UV–vis spectra of NiOESPz.

**Figure 4:**
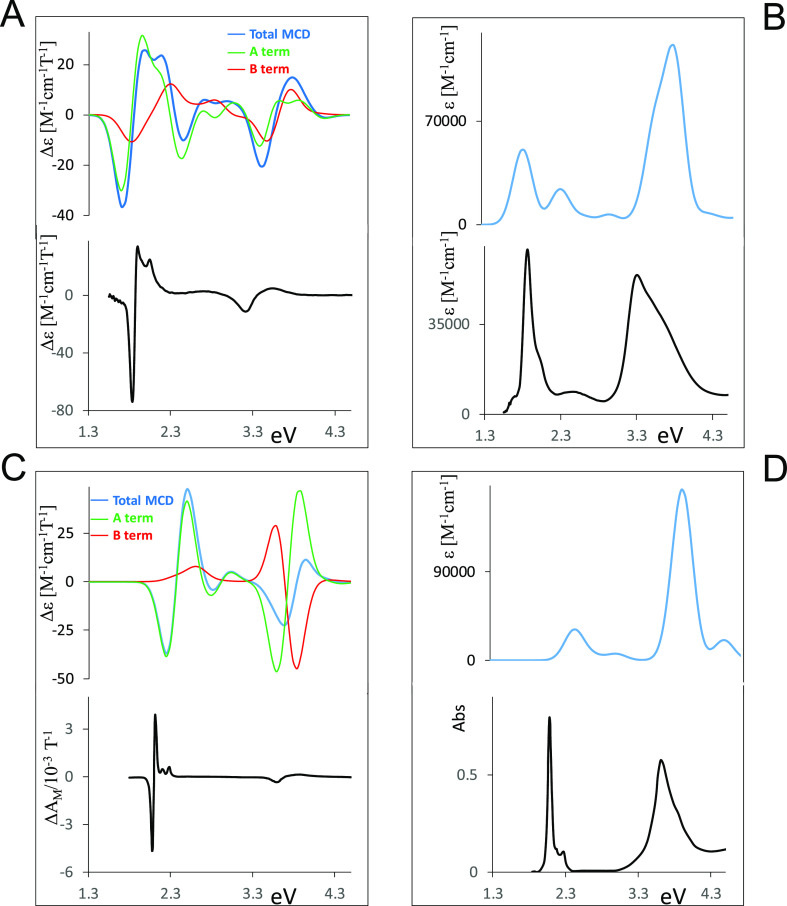
Comparison of experimental (black lines) and calculated (colored lines) UV–vis and MCD spectra. (A) Calculated and experimental MCD spectra of MgOESPz. (B) Calculated and experimental UV–vis spectra of MgOESPz. (C) Calculated and experimental MCD spectra of MgOEPz. (D) Calculated and experimental UV–vis spectra of MgOEPz.

From the performed calculations, it is possible to evaluate the spectroscopic response also. A comparison of experimental and calculated spectra is given in Figure 3 for the “free base” and for NiOESPz and in Figure 4 for MgOESPz and MgOEPz. The correspondence is not perfect, but acceptable, and allows us to recognize and to assign the various features, as reported in Table 1.

**Table 1:**
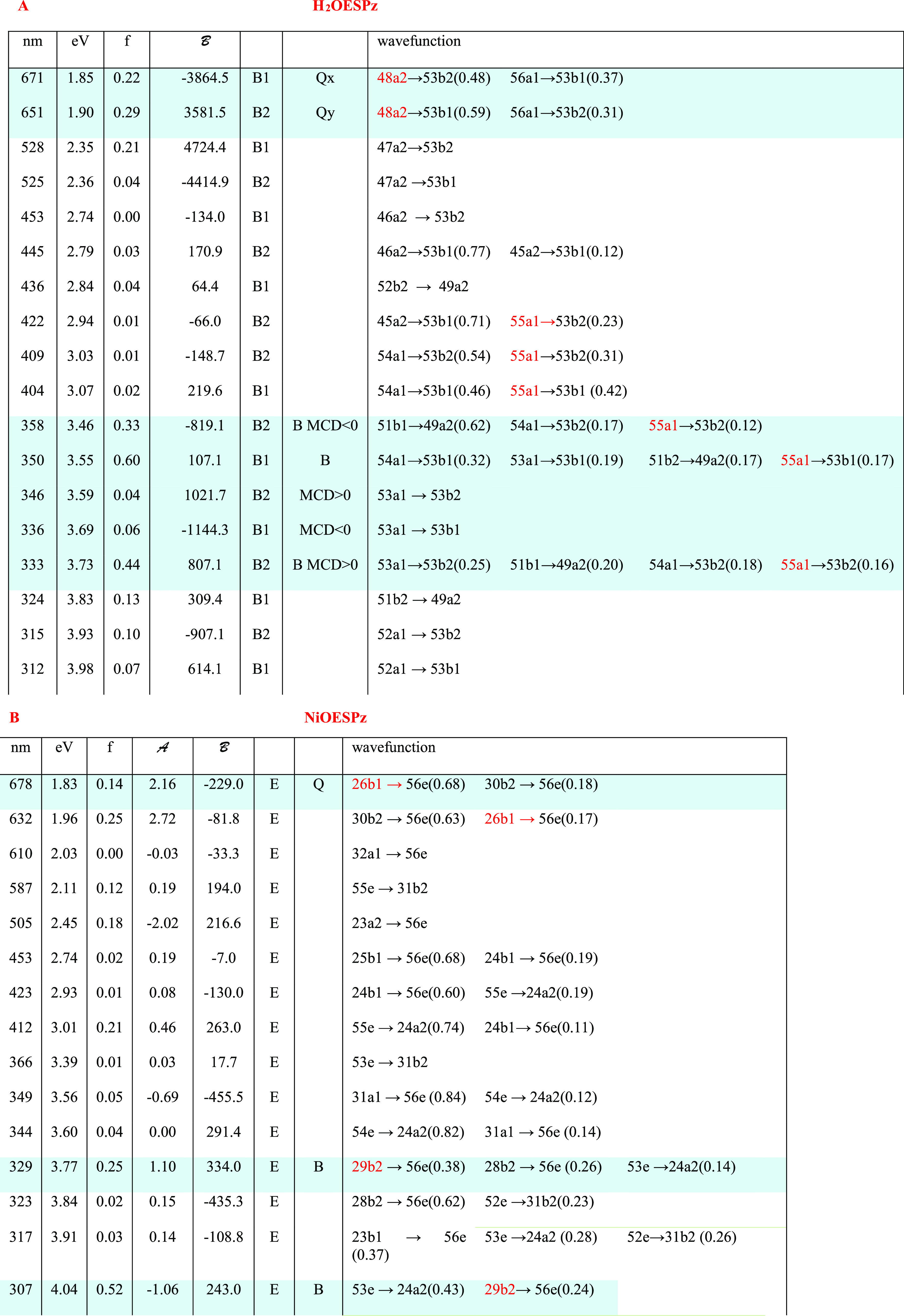

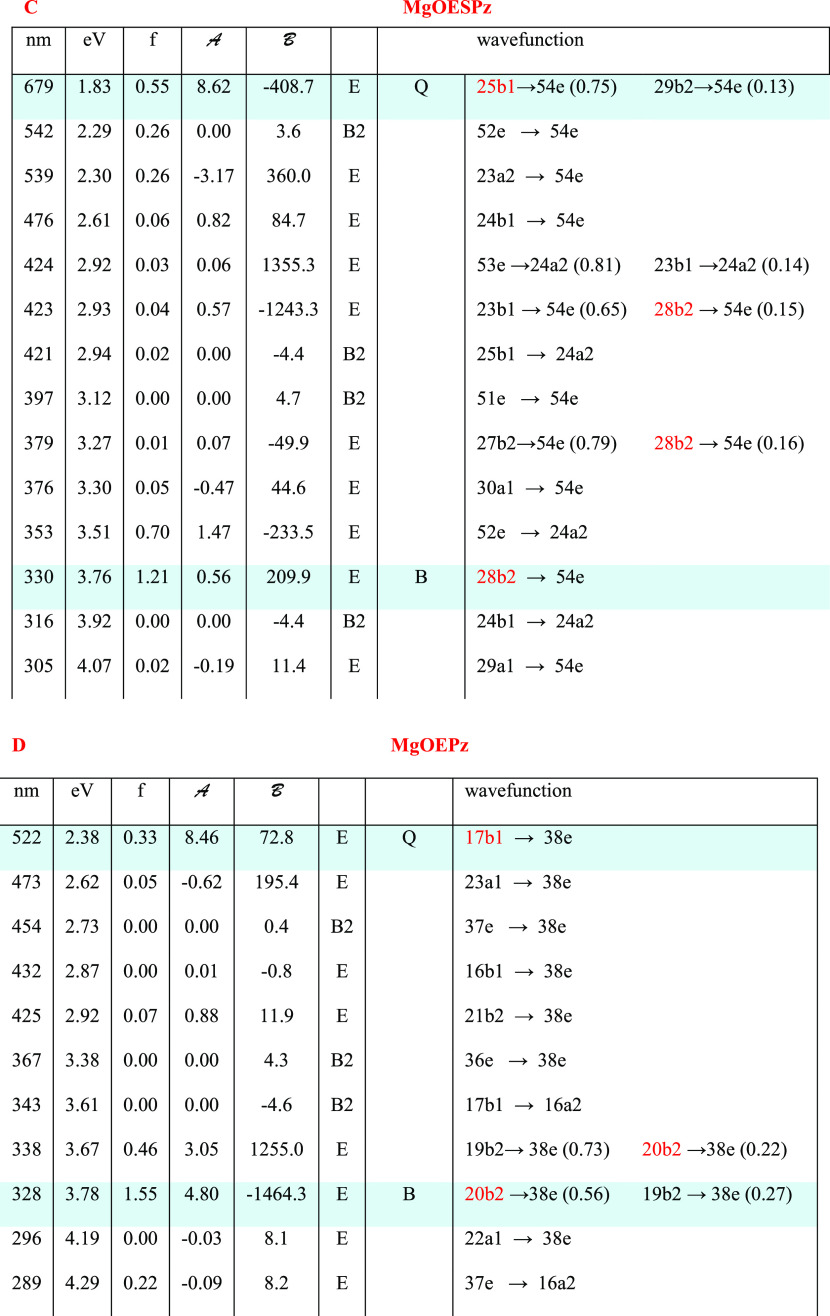
Principal Calculated Transitions Accounting for the Observed Bands: Wavelength (nm), Energy (eV), Oscillator Strength (f), Magnetic Terms A (au) and B (au), Symmetry, and Wavefunctions^a^

a Occupied Gouterman orbitals are written in red.

The two Q*_x_*and Q*_y_* components observed for the metal-free compound can be assigned to transitions from 48a2, the HOMO Gouterman orbital bearing high contributions from sulfur atoms to LUMO and LUMO + 1 states. They possess similar oscillator strengths and two B-terms, the lowest energy one negative and the second positive, as expected, since the two HOMO, HOMO – 1 Gouterman states present an energy difference value larger than that for LUMO, LUMO + 1.^34,35^ The intense B band receives contributions from three main transitions evidenced in Table 1A, involving the pure Gouterman type orbital 55a1 only partially and also presenting consistent contributions from orbitals of the same symmetry but involving sulfur atoms, like 54a1. As already observed, absorption and MCD features are also recorded and calculated at intermediate energies between the Q and B regions. The main absorption contributions at about 2.35 eV (528 nm) involve the occupied orbital 47a2 localized on sulfur atoms and present B-terms of the opposite sign, partially canceling out due to near-degeneracy. The positive MCD feature observed at about 2.85 eV (435 nm) is calculated as the convoluted sum of many transitions between 2.73 and 3.07 eV (454, 404 nm), with contribution from orbitals quite delocalized on the azaporphyrin core and on the sulfur pendant groups and also with some contribution from the Gouterman orbital 55a1. It should be noted, however, that the features calculated in this intermediate range are highly dependent on the pendant groups’ conformation (which also dictates different symmetries). A comparison with the spectra calculated for the *D*_2_*udud* conformation is shown in Figure S4.

The absorption and MCD spectra of the nickel complex are similar to those of the “free base”. From the results of the calculations in Table 1B, one may see that the first two transitions are separated by only 0.13 eV (45 nm), and present two positive A-terms giving rise to a spectral shape similar to the first set of four transitions for the “free base”, for which they exhibit a large oscillator strength and a sequence of (-,+) (-,+) B-terms. In the nickel case, however, the long positive tail bears contribution from a transition calculated at 2.45 eV (506 nm) with the negative A-term; in this case the positive low-energy contribution sums up with the higher energy positive contribution of the positive A-term of the previous transition. B-terms are present but show minor relevance. The nickel (32a1) → LUMO metal to ligand transition has a negligible oscillator strength and MCD activity. The intense B band receives contributions from the two main transitions evidenced in Table 1B, partially involving the Gouterman type orbital 29b2 and presenting consistent contributions also from sulfur atoms (53e → LUMO + 1 in the case of the second intense transition); these two transitions show a positive and a negative A-term, respectively. However, also B-terms are to be considered in this region, as illustrated in Figure 3C. It should be noted that in the presence of Ni, the B band is blue-shifted when compared with the MgOESPz complex or metal-free H_2_OESPz and the same is observed with the Cu metal complex. Absorption and MCD features are present in between the Q and B regions and in this case, the contribution of B-terms appears responsible for the MCD activity.

Similar comments/assignments can be made for MgOESPz (Figure 4). The MCD spectrum is characterized by a doublet with a peculiar shape, which can be assigned to an A-term involving Gouterman orbitals with a non-negligible negative B contribution, followed by a second transition with an initial state 52e localized on sulfur atoms, with a smaller oscillator strength, opposite A-term, and opposite B term (Table 1C).

The intermediate spectroscopic region corresponds to absorption with quite a low oscillator strength and MCD activity; two degenerate intense B-terms of opposite signs nearly cancel each other. The B band presents two components; the one with a larger oscillator strength, at higher energy, can be assigned to the classical Gouterman orbital 28b2, while the one bearing the largest MCD activity is due to a positive A-term again involving sulfur contributions of the initial state 52e and the excited state 24a1. Considering the band assignment for the three cases just examined, we can say that the configuration interaction evidenced a complex pattern underneath the high-energy B features and the weak “extra” band W; the classical HOMO – 1 Gouterman orbital is well localized on the macrocycle nitrogen atoms, even though it contributes to the B band together with orbitals localized on sulfur atoms; orbitals with different symmetries contribute to the “extra” band transitions with just a weak involvement of the HOMO – 1 orbital. Through the same type of calculations, energy levels, wavefunctions based on eigenvectors predicted by the TDDFT method, absorption, and MCD spectra of the octaethyl Mg porphyrazine complex can be obtained, and the findings are very similar to the other literature studies on porphyrazines. MgOEPz presents few sharp bands calling for a much simpler picture; all interesting orbitals (Figure S3B) are localized on the core and the Gouterman orbitals, not perturbed by other heteroatoms, are in accord with the picture already discussed for porphyrazine, with large MCD contributions in correspondence to the Q band, and quite small ones for the B band. In detail, as seen in Table 1D, the MCD spectrum is well accounted for by positive A-terms; important B-terms are calculated in correspondence to the B region but they are opposite and nearly degenerate, so they nearly cancel each other. Notice that aiming to compare MgOESPz and MgOEPz orbitals to evidence sulfur contributions, we maintained the *uudd* conformation also for the MgOEPz complex, despite the fact that it is not the lowest energy one and it possesses lower symmetry than the usual one *udud*. A similar comparison has been conducted in ref (78) with octaethyl pendants, but in that case two different symmetries have been considered for the alkyl and for the thioalkyl cases.

Considering that the MCD spectra were recorded in solution, one cannot exclude the participation of other conformers whose calculated populations depend also on the adopted DFT method, particularly with alkyl and thioalkyl pendants (by the way, BP86/TZVP by the Gaussian package gives similar energy values for *uudd* and *udud* conformers of MgOESPz, the first one with a slightly lower value; considering instead BP86/TZP calculations with the ADF *uudd* conformer is more stable such that the calculated *udud* conformer population is negligible). As an example, we report here both conformers for MgOESPz to analyze the effect on A- and B-terms (see Figure S4). It is interesting to notice that the final result (i.e., A + B) is quite similar in shape for the two cases; only some wavelength shift is observed, which can at least partially explain the band broadness observed for these compounds; the detailed contributions from A and B instead are quite different, the major differences being observed in the high-energy spectroscopic range, where many orbitals interact to give the “bright” transitions. Obviously, the details of the orbital description and band assignment are different in the two cases since sulfur *n* orbitals alternate their orientation in a different way in the two cases, dictating the different symmetry.

### Discussion of an Open-Shell System (CuOESPz)

**Figure 5:**
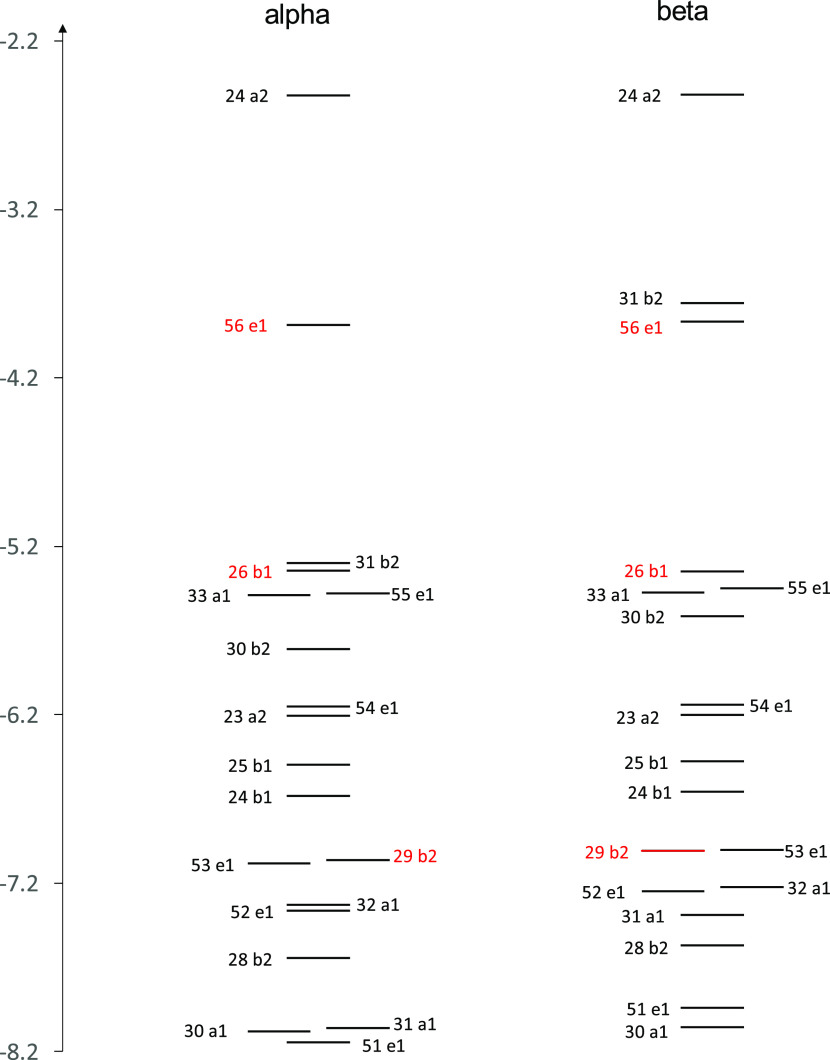
Calculated energy levels CuOESPz (Gouterman orbitals written in red).

A similar TDDFT analysis as the one presented above, can be conducted also for open-shell systems,^80−82^ and we did it for the CuOESPz complex. α and β energy levels are reported in Figure 5, where we highlighted Gouterman orbitals 26b1 and 29b2 (in red) and MO derived primarily from the d*_z_*_^2^_ orbital of the central metal β 31b2 (unoccupied) and β 32a1 and α and β 31a1. The latter orbitals are represented in Figure S5; from performed calculations, metal to ligand transitions present negligible contributions to absorption and MCD spectra, see Table S1 for details.

**Figure 6:**
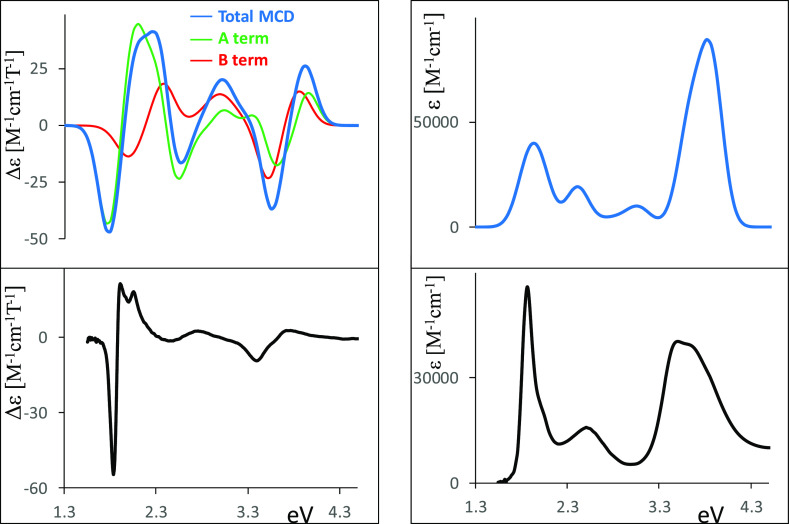
Comparison of experimental (black lines) and calculated (colored lines) UV–vis and MCD spectra of CuOESPz.

The resulting calculated MCD and absorption spectra are reported in Figure 6, and give acceptable results as compared with the previously examined closed-shell cases; in particular, transition energies are in good correspondence to the observed bands. The shape of the doublet observed in the Q region, similar to what was observed for the Mg complex, is again explained by a negative A-term (at 2.41 eV = 514 nm), which in this case follows two nearly degenerate positive A-terms, calculated at 1.86 (667 nm) and 2.01 eV (614 nm).

### Coordination Chemistry of [Mn(OESPz)(SH)]

As anticipated in the introduction, the thioethyl porphyrazine Mn(II) complex, MnOESPz, possesses peculiar properties at the nucleophilic metallic center compared to the congener Mn(II) tetrapyrroles.^55−58^ In fact, it promotes the easy removal of halogen atoms from halogenated hydrocarbons, via oxidative addition of the halogen to manganese. Moreover, the Mn(II) thioethyl porphyrazine complex, MnOESPz, reacts also toward weak electrophiles like carbon disulfide to afford the hydrosulfide derivative [Mn(OESPz)(SH)] (Scheme 1).^56^ This system deserves particular interest from the biological point of view because transition-metal tetrapyrroles with a hydrosulfide axial ligand mimic the spectral properties of cytochrome P-450 and chloroperoxidase^83^ and mono- and polynuclear Mn(III) are of central importance in biological systems such as superoxide dismutase^84,85^ and catalase.^86^ The coordinating capability of the [Mn(OESPz)(SH)] complex is also investigated by titration with a strong unhindered σ-donor base like 1-methylimidazole (1-mim), observing that 1-mim in chloroform coordinates with manganese giving rise to a [Mn(OESPz)(SH)(1-mim)] complex, while in benzene solution, upon addition of 1-mim, a redox Mn(III)/Mn(II) process occurs leading to the formation of a Mn(II) species.^56^ Such titration, previously monitored by UV–vis spectroscopy, is repeated herein (vide infra) following the process via MCD spectroscopy and considering a wider 1-mim concentration range.

**Figure 7:**
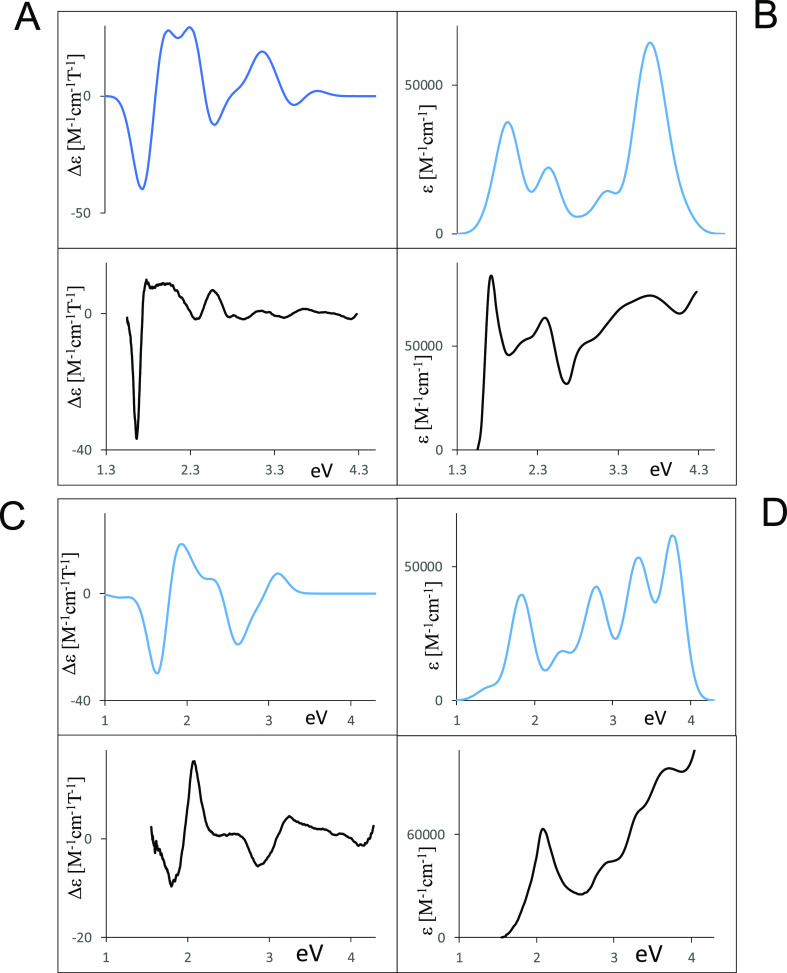
Comparison of experimental (black lines) and calculated (blue lines) UV and MCD spectra of the manganese complex. (A) Calculated and experimental MCD spectra of [Mn(OESPz)(SH)]. (B) Calculated and experimental UV–vis spectra of [Mn(OESPz)(SH)]. (C) Calculated and experimental MCD spectra of [Mn(OESPz)(1-mim)_2_]. (D) Calculated and experimental UV–vis spectra of [Mn(OESPz)(1-mim)_2_].

The initial complex, [Mn(OESPz)(SH)], is characterized by a square pyramidal geometry and total spin *S* = 2.^55−58^ Its recorded MCD spectrum (Figure 7A) presents two opposite B-terms associated with Q*_x_* and Q*_y_* transitions, with a negative signal at 1.67 eV (742 nm) and a positive signal at 1.82 eV (681 nm), the latter one characterized by an evident shoulder. Moving to higher energy, a weak negative feature and an intense positive one are observed, in correspondence to the absorption peak centered at 2.39 eV (519 nm). The Soret region shows several non-well-defined weak MCD signals, while the UV–vis spectrum shows an intense broad absorption. UV–vis and MCD spectra of the [Mn(OESPz)(SH)] complex is simulated by TDDFT calculations (Figure 7A,B). Overall, the main features of MCD and UV–vis spectra are well reproduced, even if the calculated signals are computed at a slightly higher energy.

The Q*_x_* and Q*_y_* components can be assigned to transitions from the occupied Gouterman HOMO state to LUMO and LUMO + 1 Gouterman states (α 228 → 232 or β 226 → 228 and α228 → 233 or β 226 → 229), as reported in Table 2. The corresponding orbitals are reported in Figure S6, one may observe that the −SH ligand contributes to the LUMO and LUMO + 1 orbitals. The absorption band calculated at 2.39 eV (519 nm) has a n → π* (α 223 → 232) component involving nitrogen and sulfur atoms of the thioalkyl chains. At a higher energy, we calculate transitions between 3.02 eV (411 nm) and 3.09 eV (401 nm) with some HOMO Gouterman orbital β 226 contributions. At 3.45 eV (359 nm), the B band region, a high oscillator strength and a negative B term are calculated in correspondence to a combination of transitions involving the occupied orbitals α 217a, β 215a (Gouterman orbitals), and α 213a with high contributions from the metal and −SH ligand. Other orbitals (not shown in Figure S6) are localized on Mn or Mn-SH, however, they do not contribute to “bright” transitions.

**Table 2:**
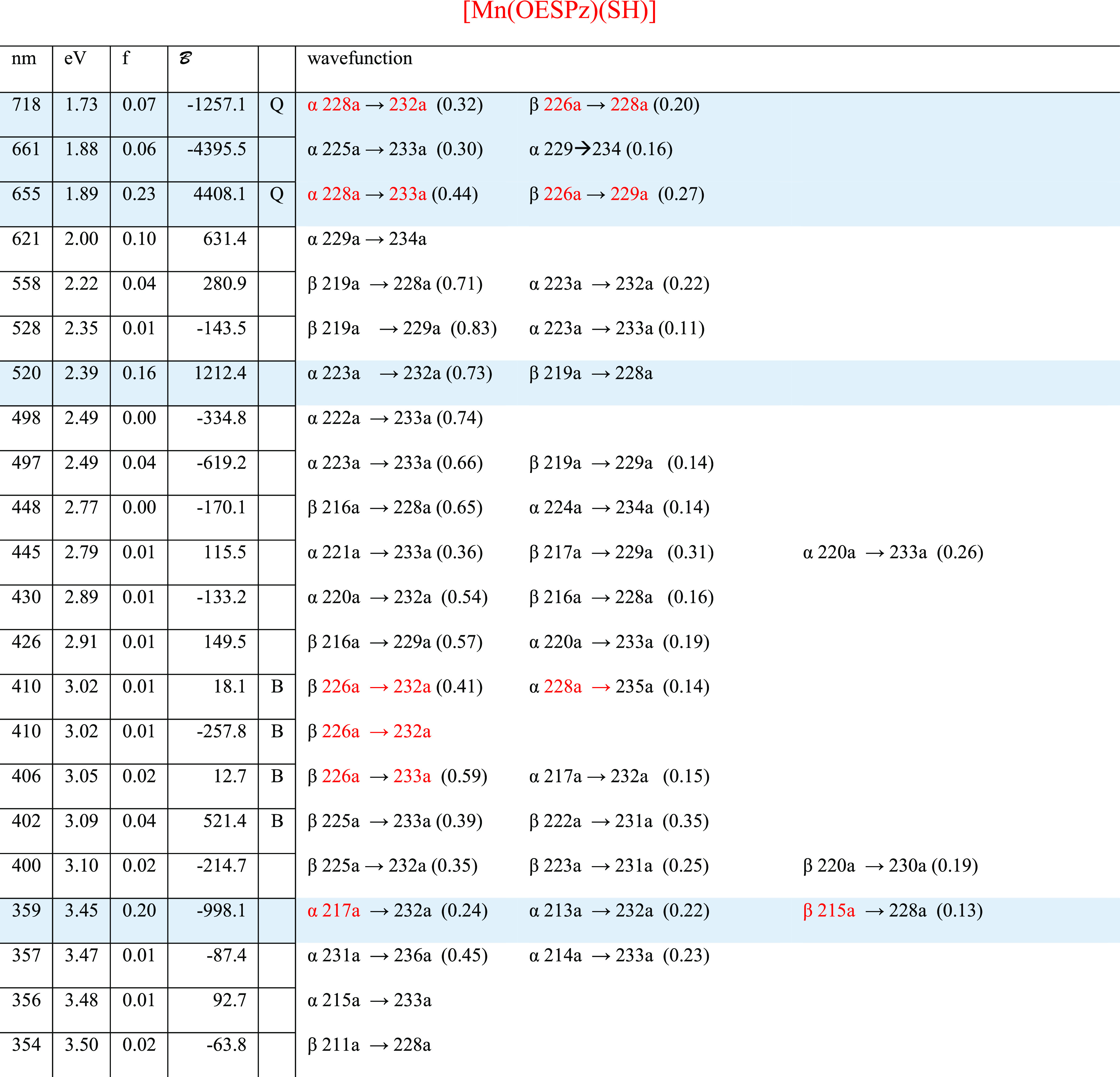
Principal Calculated Transitions Accounting for the Observed Bands: Wavelength (nm), Energy (eV), Oscillator Strength (f), Magnetic Terms A (au) and B (au), and Wavefunctions^a^

a Gouterman orbitals are written in red.

**Figure 8:**
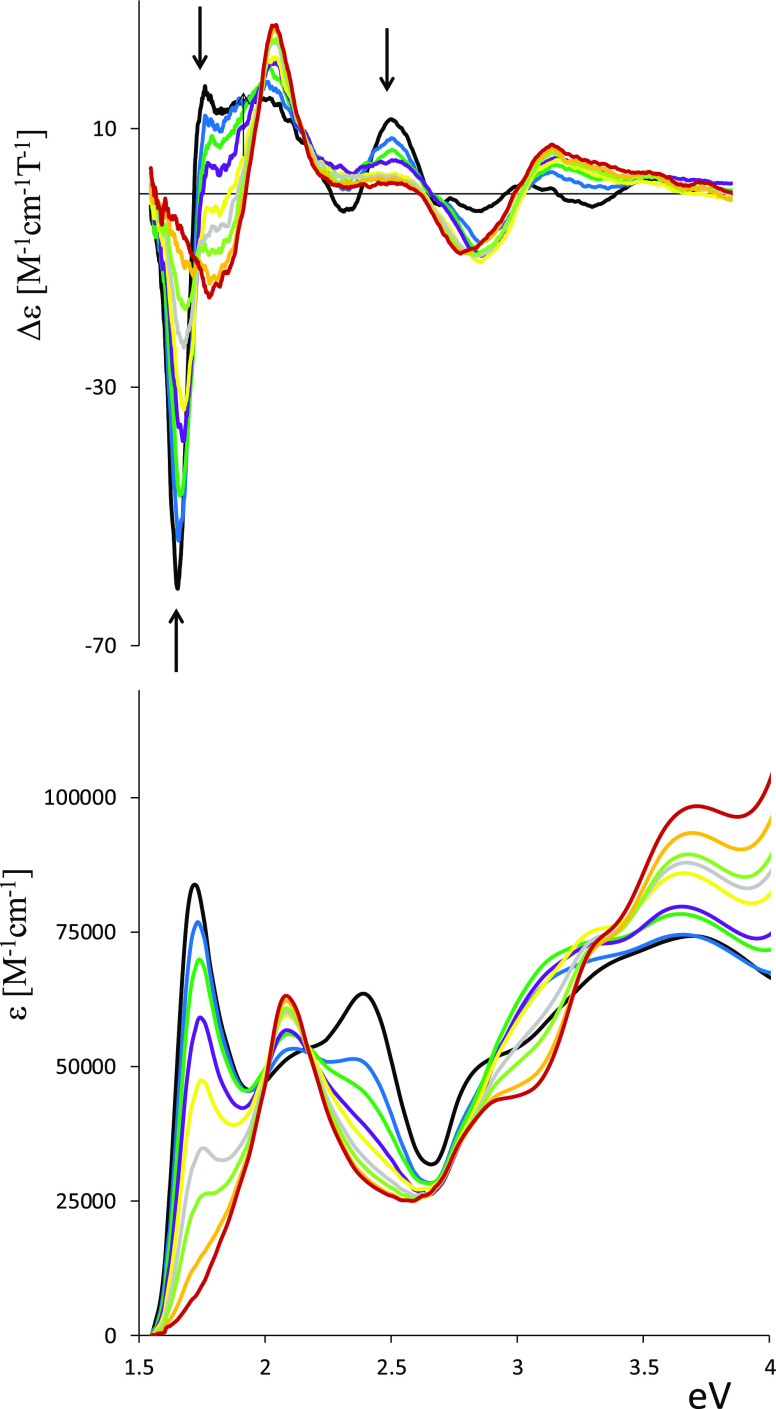
Spectroscopic titration of 0.012 mM solution of [Mn(OESPz)(SH)] with 1-mim (0–0.294 M) in benzene. Black = no 1-mim and red = highest 1-mim concentration.

Treatment of a dilute (0.012 mM) benzene solution of [Mn(OESPz)(SH)] with aliquots of 1-mim in the same solvent, under rigorous anaerobic conditions, leads to isosbestic changes in both the UV–vis and MCD spectra (Figure 8). At relatively high concentrations of the nitrogenous base, limiting spectra indicative of a Mn(II) species was obtained. The observed final MCD spectrum shows a minus–plus signal in the Q region, shifted at a higher energy with respect to the signal of the initial complex [Mn(OESPz)(SH)], in correspondence to the absorption at 2.05 eV (604 nm).

The UV–vis at high energy presents an overlapping of many features, three main components are observed at 2.92, 3.31, and 3.65 eV (425, 375, and 340 nm, respectively). By plotting ln[(Δε_i_ – Δε)/(Δε −Δε_f_)] versus ln[1-mim] at various wavelengths^87−89^ (see Figure S7), an approximately linear plot is obtained with a slope of 2 ± 0.1, identifying the reaction as complexation of 2 equiv of the axial ligand 1-mim. The almost isosbestic changes occurring in the visible spectrum may indicate that the possible monoligand intermediate [Mn(OESPz)(SH)(1-mim)] complex is present only in negligible amounts.^89^ A similar behavior has been reported for iron(III) porphyrazines,^89^ porphyrins,^90,91^ and protoporphyrins^92^ when treated in nonpolar solvents with strong field ligands (imidazoles and pyridines). Taking into account the recently reported inner-sphere reduction mechanism of Fe^III^ porphyrin complexes with hydrosulfide ligands,^93^ we can hypothesize that the Mn^III^ → M^II^ reduction process occurs with oxidation of HS^–^ to elemental sulfur through a two step sequence as reported in eqs 1 and 2. In the first step (eq 1), the reaction proceeds via homolytic cleavage of the Mn^III^–SH bond, releasing a hydrosulfide radical (HS^•^). Subsequently (eq 2) this hydrosulfide radical (SH^•^) reduces another Mn^III^ porphyrazine to a Mn^II^ complex forming elemental sulfur and releasing a molecule of H_2_S.
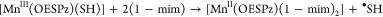
1
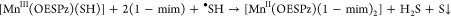
2

This mechanism resembles the biological mechanism of cytochrome *c* reduction by H_2_S^94^ and, in general, the interaction with heme centers, where 1-mim can mimic hystidine axial coordination.^95,96^

To confirm the formation of the hexacoordinate Mn(II) complex [Mn(OESPz)(1-mim)_2_] suggested by titration analysis, we decided to simulate by TDDFT calculation its UV–vis and MCD spectra. From the analysis reported in ref (58), Mn(II)OESPz has a 5/2 spin in the crystal, where it is axially coordinated to two neighbor sulfur atoms, while in solution the tetra-coordinated complex is supposed to have *S* = 3/2. For [Mn(OESPz)(1-mim)_2_], we considered both spin conditions (as presented in Figure S8). For the *S* = 5/2 case, the succession of MCD features is correctly represented (i.e., the minus–plus doublet in the Q region and the sign of the B-terms in Q and Soret regions), however, the calculated transition energies are poorly reproduced, in particular the Q band excitation energy is strongly underestimated (Figure 7C,D). The *S* = 3/2 complex, instead, does not fit the observed features at all (Figure S8). The two possible orientations of the 1-mim groups (parallel and perpendicular) have been considered and show similar calculated spectra. Finally, also [Mn(OESPz)(SH)(1-mim)] has been calculated for comparison, as presented in Figure S8. In this case, the calculated absorption spectrum is similar to the recorded one, but the calculated MCD spectrum does not match the experimental data; on the contrary the [Mn(OESPz)(1-mim)_2_] calculated MCD spectrum well fits the experimental one apart from the energy shift. These findings strongly suggest the formation of the [Mn(OESPz)(1-mim)_2_] complex upon 1-mim titration of [Mn(OESPz)(SH)] in benzene, thus clarifying the nature of the Mn(II) species hypothesized in ref (56).

We repeated the calculations of [Mn(OESPz)(1-mim)_2_] and [Mn(OESPz)(SH)] using the SAOP and B3LYP functional, but we did not obtain any improvement of the representation of the Q transition energies. It is worth recalling here that high-spin open-shell systems are difficult to treat with the linear response formulation of TDDFT;^48,49,97,98^ methods such as complete active space self-consistent field (CASSCF)^99^ and restricted active space (RAS)^100^ wavefunctions should perform better but are beyond the scope of the present work.

## Conclusions

In conclusion, the analysis of MCD and absorption data supported by TDDFT calculations clearly shows how the red shift observed for thioalkyl-substituted porphyrazine with respect to the analogous alkyl porphyrazine is due to delocalization of the molecular orbitals on sulfur atoms. In particular, sulfur atoms are involved in all bright transitions and, besides contributing to Q and B bands, give rise to the so-called W band.

Substituent thioalkyl chain conformations have an important role in determining molecular symmetry and, since sulfur atoms are heavily involved in optical transitions, A- and B-terms highly depend on the conformation. A test on the lower energy conformers with different symmetry suggests that the resulting MCD pattern A + B is however similar and follows the usual literature interpretation.

Experimental MCD spectra of tetra-coordinated metal OESPz complexes are very similar, with a few slight differences; the calculations herein presented give acceptable results; calculated band energies appear acceptable with no need for an ad hoc wavelength shift, but the B band intensity is overestimated and calculated too high relative to the Q band, which is usually better represented. Of course, simple TDDFT calculations cannot account for band shapes. Despite these limitations, differences in the wavelength of the Q band and of the B band, which are evident when comparing the metal-free, the Ni and, the Mg complexes, are reproduced by calculations (see Figure S9). TDDFT calculations show good correspondence to experimental data also for the open-shell copper complex.

Finally, the use of the MCD technique allows one to accurately monitor titration processes, in particular for the manganese complex, giving clear-cut spectroscopic evidence while varying the base concentration. TDDFT calculations have been performed for the starting complex and the expected product, while the shape of the spectra appears well reproduced, the band position is not correctly calculated. Further studies are needed to better clarify the reduced complex both from the experimental and the calculated point of view. In any case, the comparison of experimental and computed MCD spectra confirms the Mn(III) → Mn(II) redox process that interconverts the [Mn(OESPz)(SH)] complex to the [Mn(OESPz)(1-mim)_2_] species upon 1-mim treatment in benzene, and the sensitivity of MCD to the spin state suggests the presence of a high spin state (*S* = 5/2).

## Supporting Information Available

Superposed experimental UV/MCD spectra (Figures S1 and S2); molecular orbitals (Figures S3, S5, and S6); superimposed calculated spectra of conformers *uudd* and *udud* (Figure S4); plots of ln[(Δε_i_ – Δε)/(Δε – Δε_f_)] versus ln[1-mim] at various wavelengths (Figure S7); comparison of calculated spectra of high-spin and low-spin Mn-complexes (Figure S8); and comparison of calculated MCD spectra of ZnOESPz, MgOESPz, and H_2_OESPZ complexes and their corresponding experimental spectra (Figure S9) (PDF)
