# Supplementary material for: Characterization of “Free Base” and Metal Complex Thioalkyl Porphyrazines by Magnetic Circular Dichroism and TDDFT Calculations
Source: J Phys Chem B. 2020 Dec 22;125(1):264–80. doi: 10.1021/acs.jpcb.0c09277 (PMC8016196; doi:10.1021/acs.jpcb.0c09277)
Supplement: Supplementary file 1 — jp0c09277_si_001.pdf [file jp0c09277_si_001.pdf]

Supporting information for

Characterization of "Free Base" and Metal Complexes Thioalkyl Porphyrazines by Magnetic Circular Dichroism and TDDFT Calculations

Simone Ghidinelli[a], Sergio Abbate[a,b], Ernesto Santoro[a,c], Sandra Belviso[c]*, Giovanna Longhi[a,b]*

[a]Dipartimento di Medicina Molecolare e Traslazionale, Università di Brescia, Viale Europa 11, 25123; Brescia, Italy.

[bc]Istituto Nazionale di Ottica (INO), CNR, Research Unit of Brescia, c/o CSMT, via Branze 45, 25123 Brecia, Italy

[c]Dipartimento di Scienze, Università della Basilicata, Viale dell'Ateneo Lucano 10, 85100 Potenza, Italy.

* Correspondence: sandra.belviso@unibas.it; giovanna.longhi@unibs.it

LIST OF FIGURES AND TABLES

Figure S1. Superposed experimental MCD spectra of ZnOESPz, MgOESPz and CuOESPZ complexes.

Figure S2. Superposed experimental MCD spectra of H_2OESPz, NiOESPz, PdOESPZ complexes.

Figure S3A. Orbital representation for H_2OESPz, NiOESPz complexes.

Figure S3B. Orbital representation for MgOESPz and MgOEPz complexes.

Figure S4. Calculated UV and MCD spectra of *uudd* and *udud* conformers of H_2OESPz and MgOESPz .

Figure S5. Orbital representation for CuOESPz.

Table S1. Principal calculated transitions accounting for the observed bands for CuOESPz.

Figure S6. Orbital representation for [Mn(OESPz)(SH)].

Figure S7. Plots of ln[($\Delta\varepsilon_i$-$\Delta\varepsilon$)/($\Delta\varepsilon$-$\Delta\varepsilon_f$] as a function of ln[1-mim] for the three different energy values indicated in Figure 8.

Figure S8. Comparison of the calculated MCD and UV spectra of [Mn(OESPz)(SH)(1-mim)], [Mn(OESPz)(1-mim)$_2$] S=3/2, and [Mn(OESPz)(1-mim)$_2$] S=5/2 complexes and the corresponding experimental spectrum.

Figure S9. Comparison of the calculated MCD spectra of ZnOESPz, MgOESPz and H_2OESPZ complexes and their corresponding experimental MCD spectra.

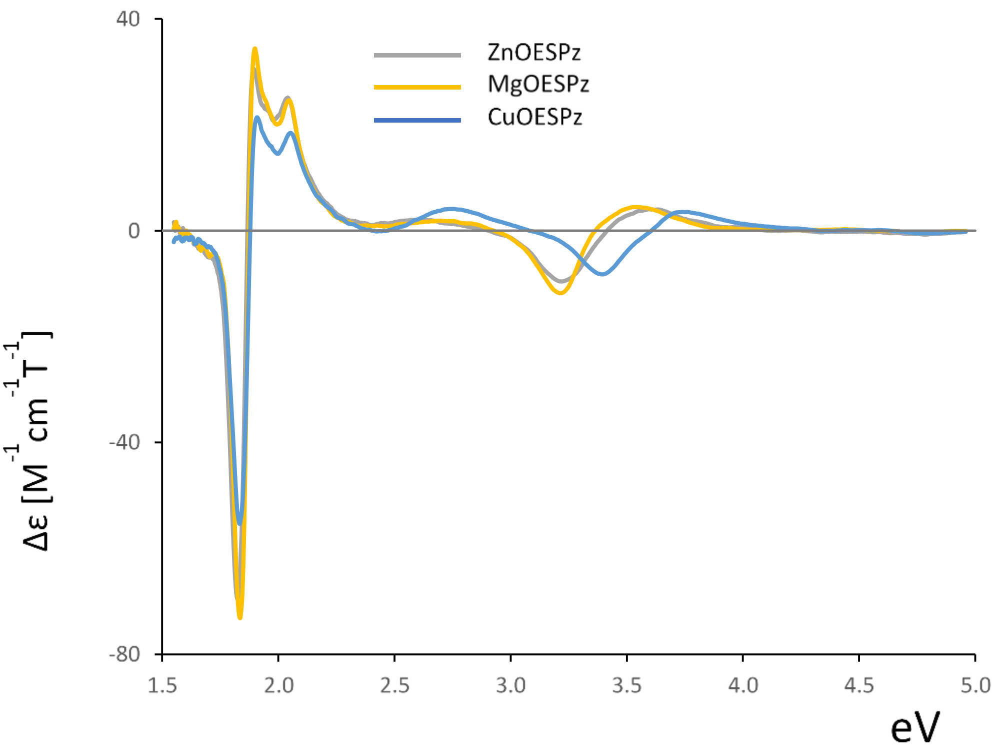

Figure S1. Superposed experimental MCD spectra of ZnOESPz, MgOESPz and CuOESPZ complexes.

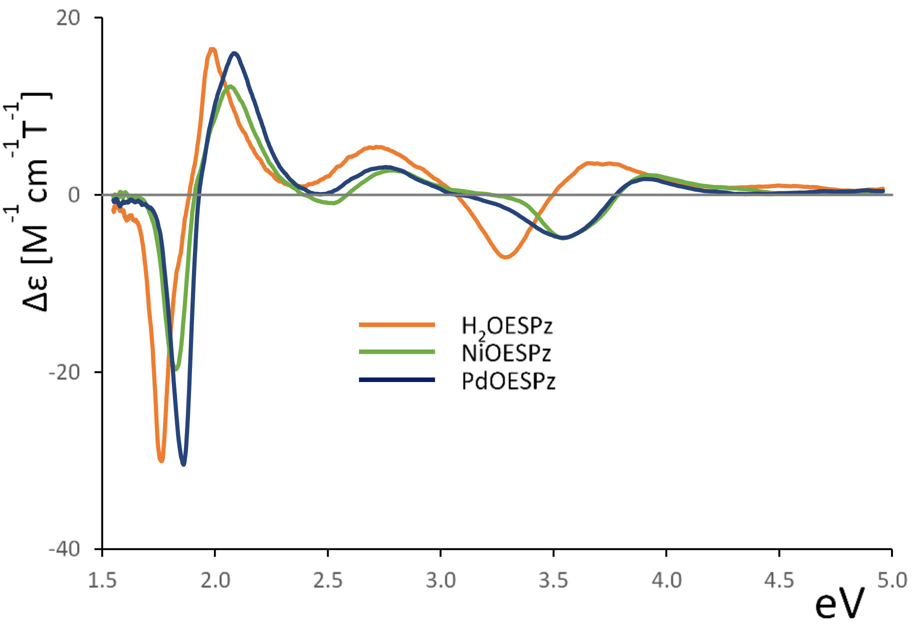

Figure S2. Superposed experimental MCD spectra of H_2OESPz, NiOESPz, PdOESPZ complexes.

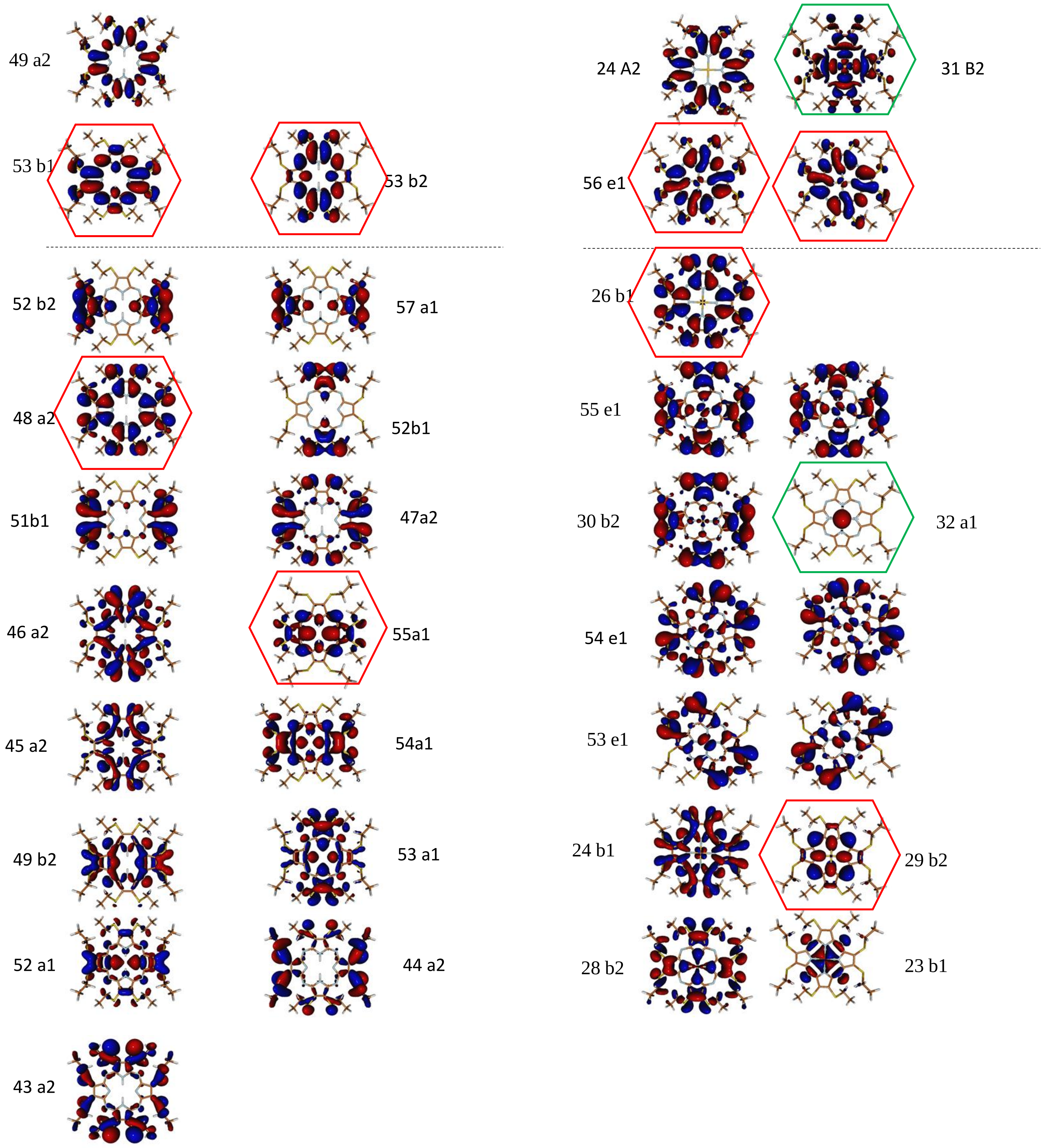

Figure S3A. Orbital representation for H_2OESPz (*left*), NiOESPz (*right*) complexes: Gouterman orbitals are evidenced in a red frame, “Nickel” orbitals in a green frame.

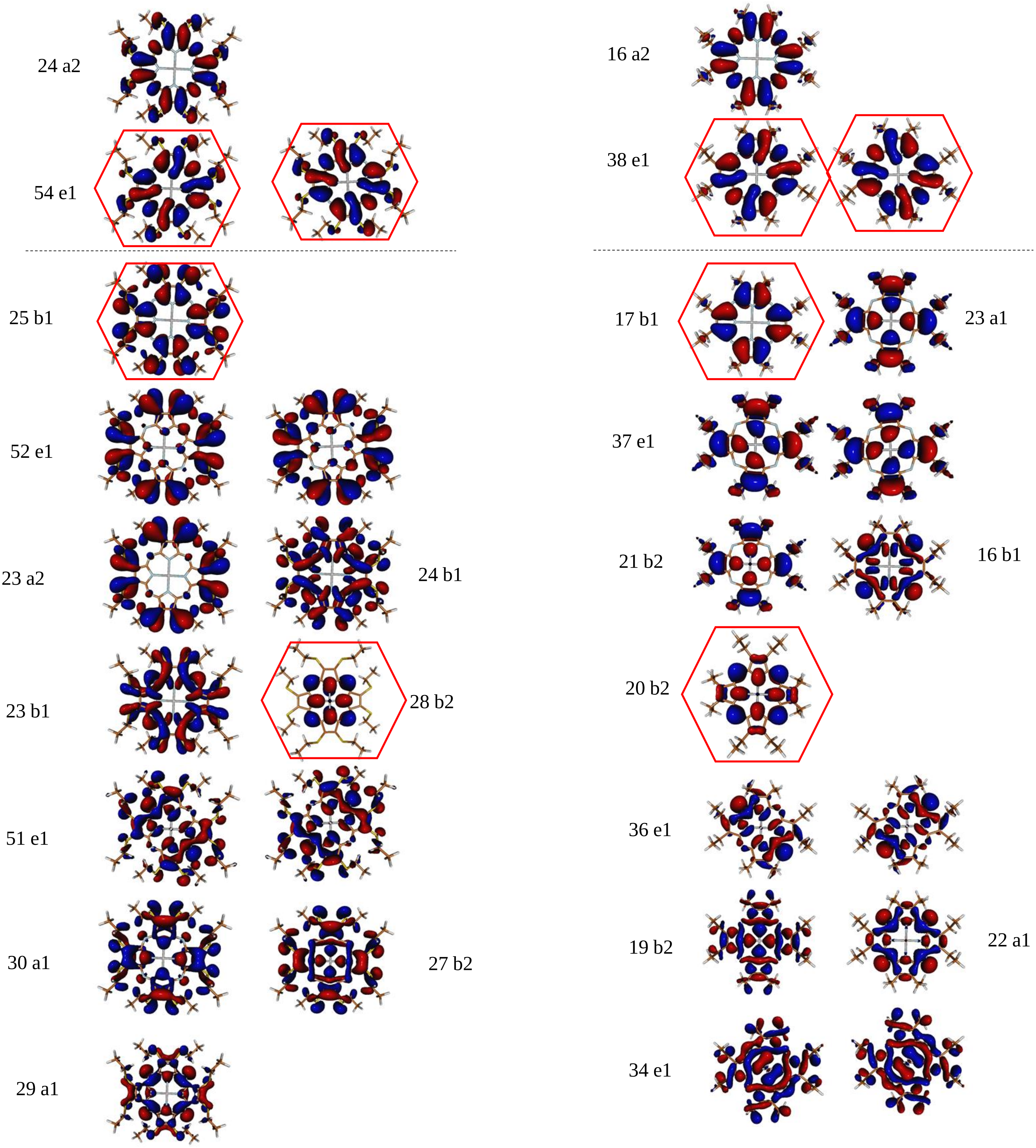

Figure S3B. Orbital representation for MgOESPz (*left*) and MgOEPz (*right*) complexes: Gouterman orbitals are evidenced in a red frame.

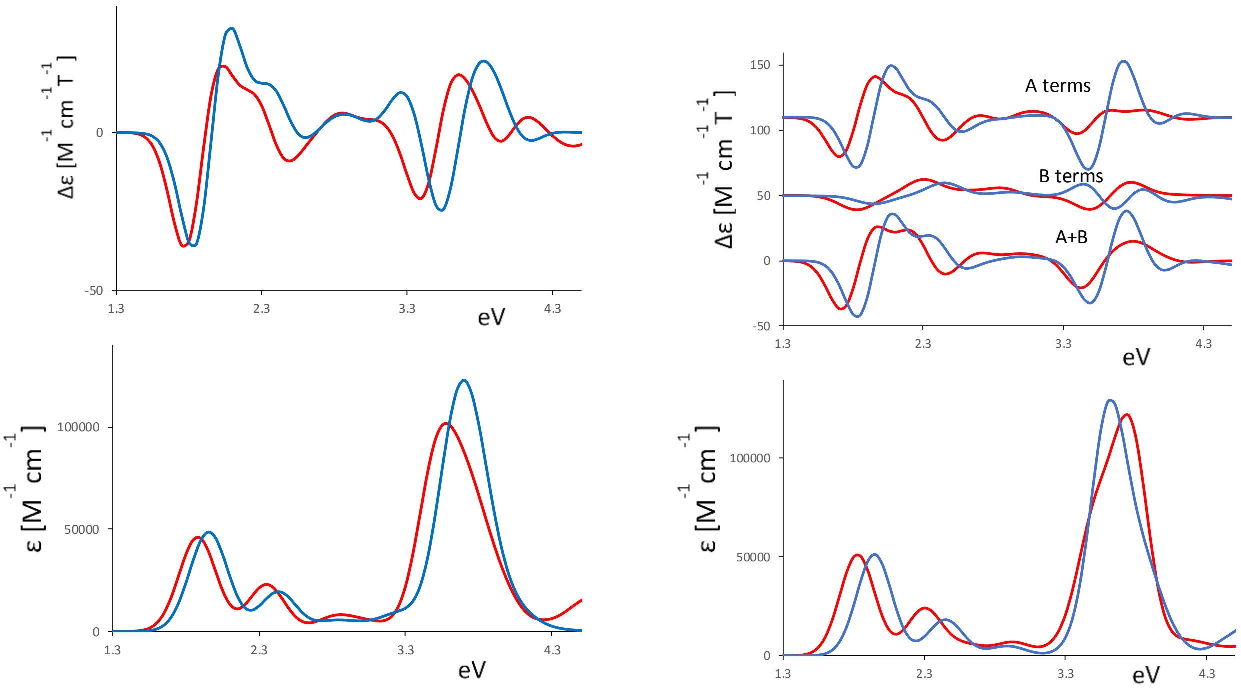

Figure S4. Calculated UV-vis and MCD spectra of *uudd* (red line) and *udud* (blu line) conformer of H_2OESPz (*left*) and , MgOESPz (*right*). For MgOESPz, the contribution of $\mathcal{B}$ terms, the contribution of $\mathcal{A}$ terms and the sum of the two contributions are shown separately.

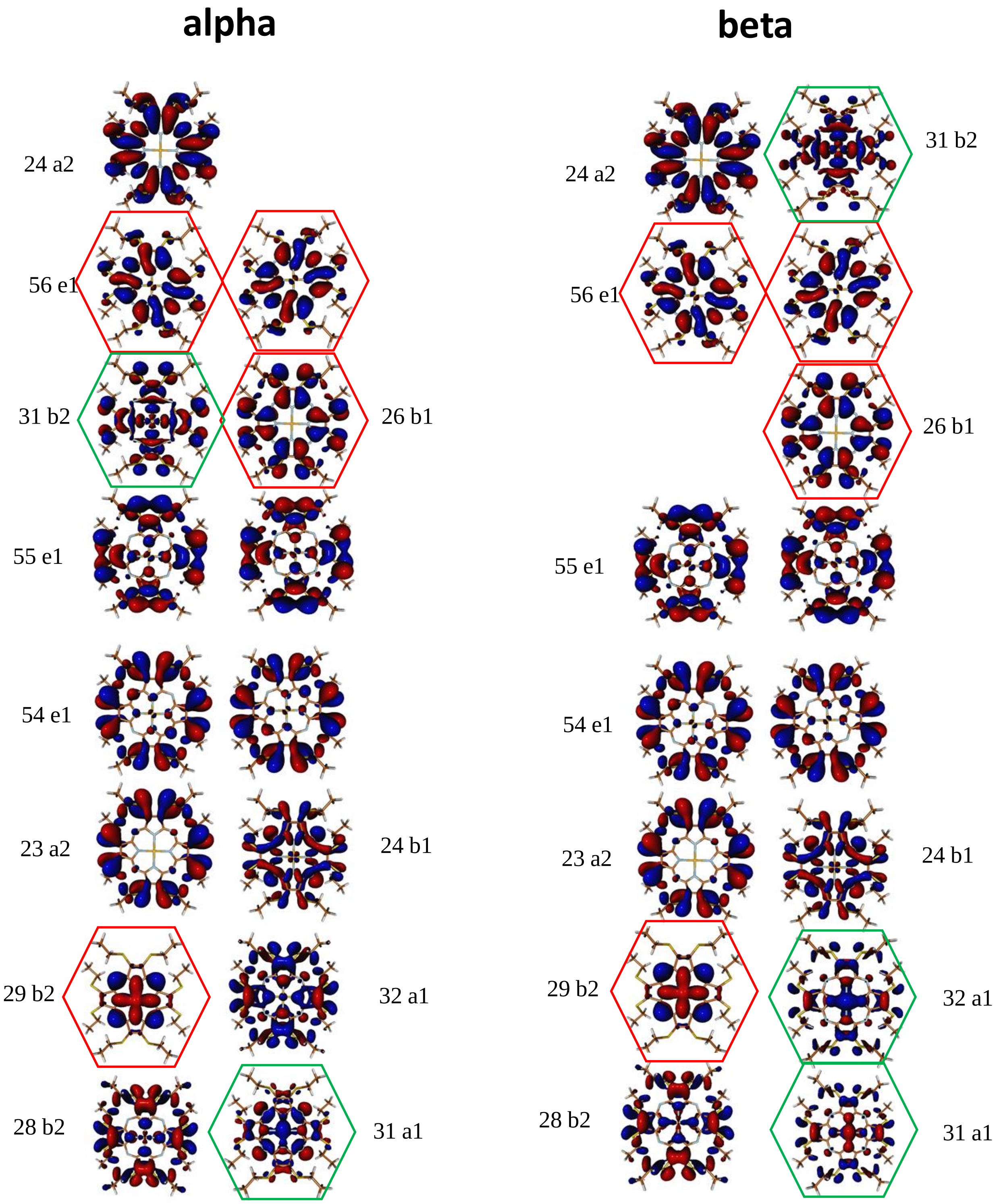

Figure S5. Orbital representation for CuOESPz. Gouterman orbitals are evidenced in a red frame; “Copper” orbitals in a green frame.

Table S1. Principal calculated transitions accounting for the observed bands for CuOESPz : wavelength (nm), energy (eV), oscillator strength (f), magnetic terms $\mathcal{A}$ (a.u.) and $\mathcal{B}$ (a.u.), wavefunctions. Occupied Gouterman orbitals are written in red.

A				CuOESPz					
nm	eV	f	$\mathcal{A}$	$\mathcal{B}$			Wavefunction		
666	1.86	0.27	9.21	-65.0	7E	Q	β 26b1→ 56e (0.34)	α 26b1→ 56e (0.31)	α 30b2→ 56e (0.21)
618	2.01	0.24	6.66	-443.3	8E		α 30b2→ 56e (0.67)	β 30b2→ 56e (0.12)	
518	2.39	0.01	-0.04	1267.4	10E		β 54e→ 31b2		
515	2.41	0.20	-4.25	-742.8	11E		β 23a2→ 56e (0.50)	α 23a2→ 56e (0.37)	
454	2.73	0.02	0.59	-8.2	13E		β 24b1→ 56e (0.42)	β 25b1→ 56e (0.29)	
451	2.75	0.01	0.31	70.9	14E		α 24b1→ 56e (0.61)	β 24b1→ 56e (0.14)	
423	2.93	0.03	1.02	179.9	16E		β 24b1→ 56e (0.39)	α 24b1→ 56e (0.34)	
403	3.07	0.08	-0.01	168.8	18E		α 55e → 24a2 (0.54)	β 55e → 24a2 (0.39)	α 29b2→ 56e (0.05)
378	3.28	0.01	-0.01	34.6	19E		β 53e → 31b2		
359	3.45	0.01	-0.17	-19.8	21E		α 32a1→ 56e (0.69)	β31a1→ 56e (0.19)	
351	3.53	0.01	-0.04	36.6	22E		β 52e → 31b2		
349	3.55	0.07	-1.22	-645.2	23E		β 31a1→ 56e (0.76)	α 32a1→ 56e (0.15)	
344	3.60	0.01	0.01	-21.5	24E		β 54e → 24a2 (0.62)	α 54e → 24a2 (0.37)	
341	3.63	0.45	1.54	299.5	25E	B	α 54e → 24a2 (0.44)	β 54e → 24a2 (0.21)	α 29b2→ 56e (0.14)
336	3.69	0.00	0.05	-66.6	26E		β 28b2→ 56e (0.75)	α 28b2→ 56e (0.23)	
326	3.80	0.00	0.07	-46.8	27E		α 28b2→ 56e (0.76)	β 28b2→ 56e (0.22)	
322	3.85	0.83	1.71	310.4	28E	B	α 29b2→ 56e (0.25)	β 29b2→ 56e (0.23)	α 54e → 24a2 (0.16)

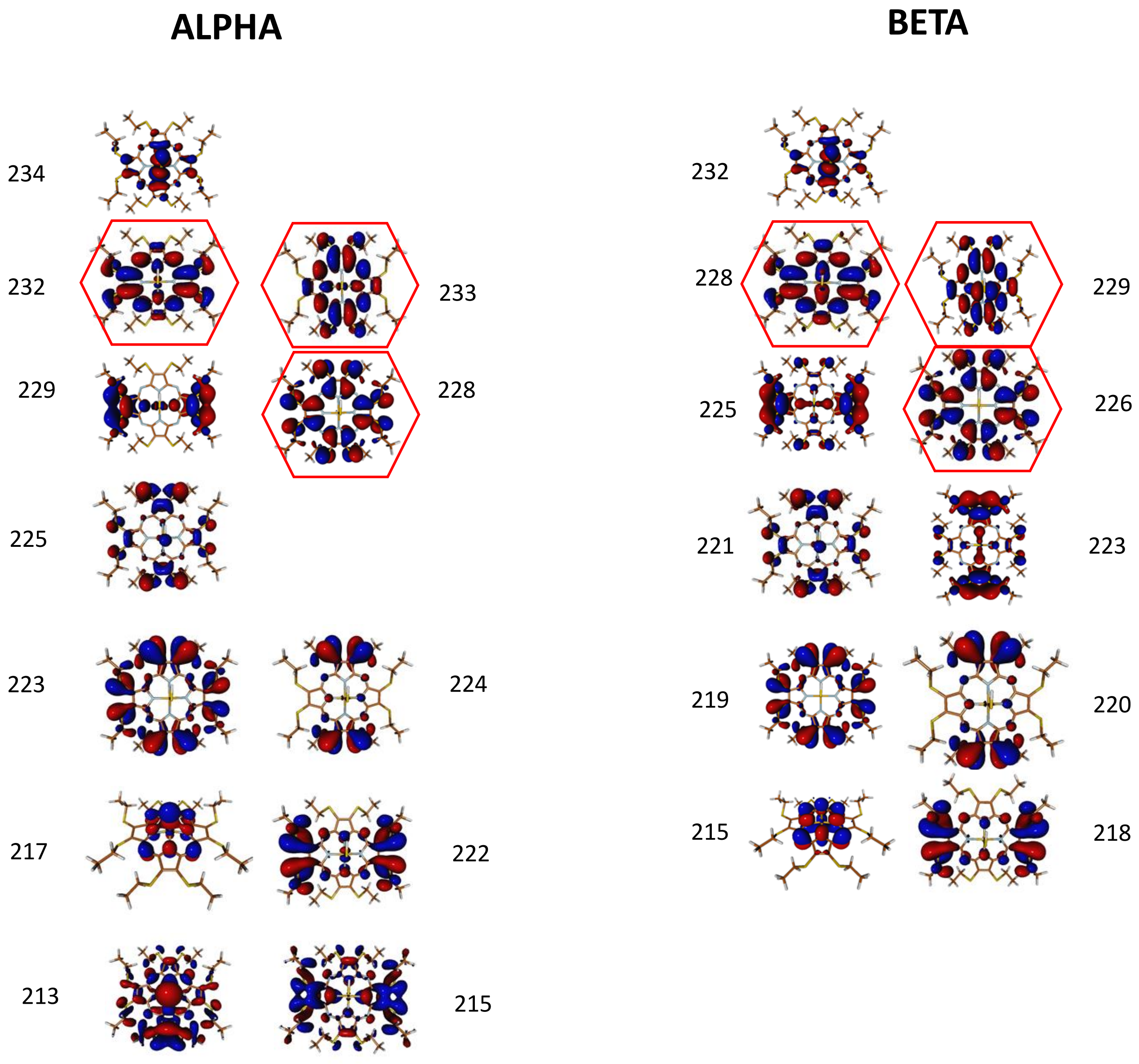

Figure S6. Orbital representation for [Mn(OESPz)(SH)]. Gouterman orbitals are evidenced in a red frame.

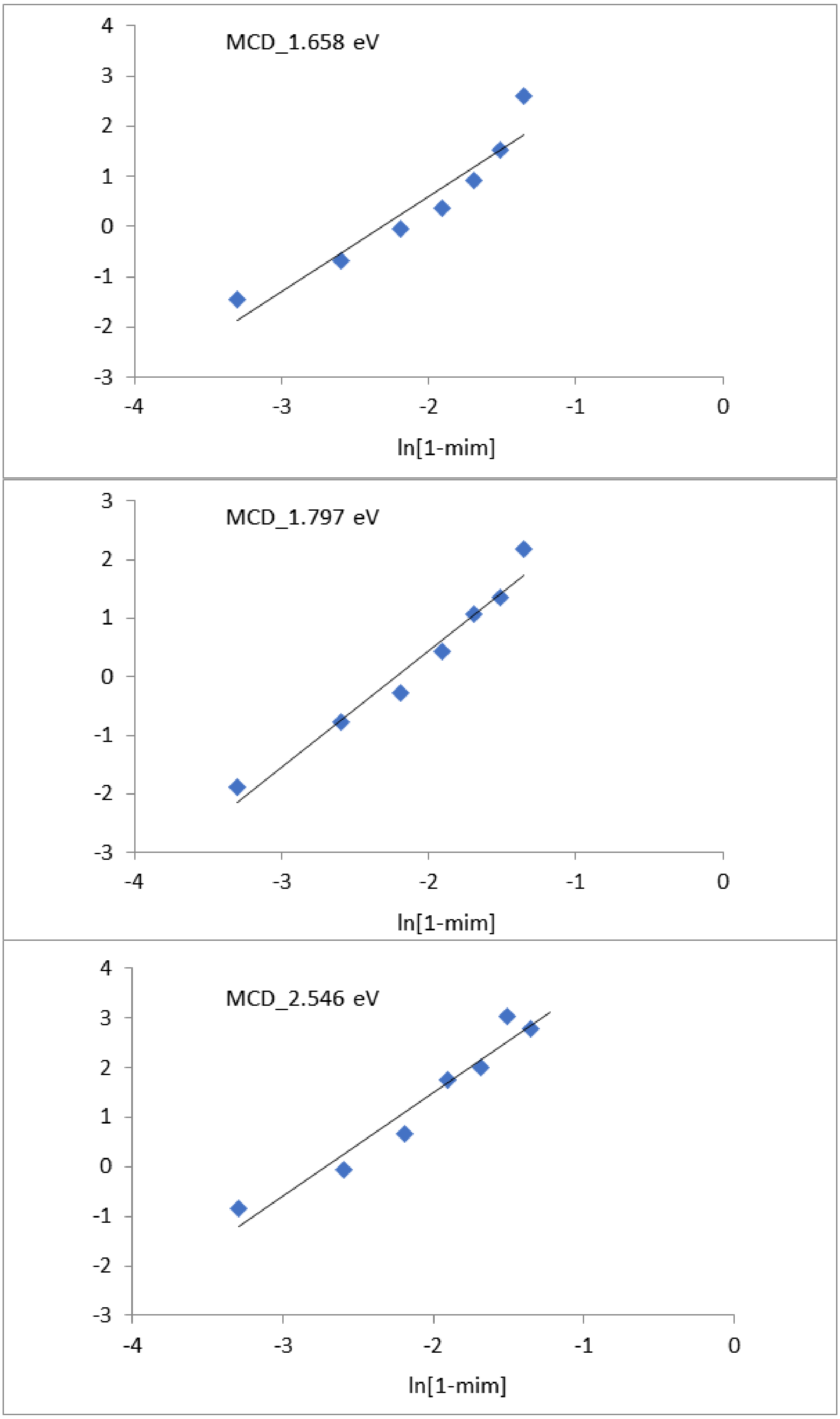

Figure S7. Plots of ln[(Δε$_i$-Δε)/(Δε-Δε$_f$] as a function of ln[1-mim] for the three different energy values indicated in Figure 8.

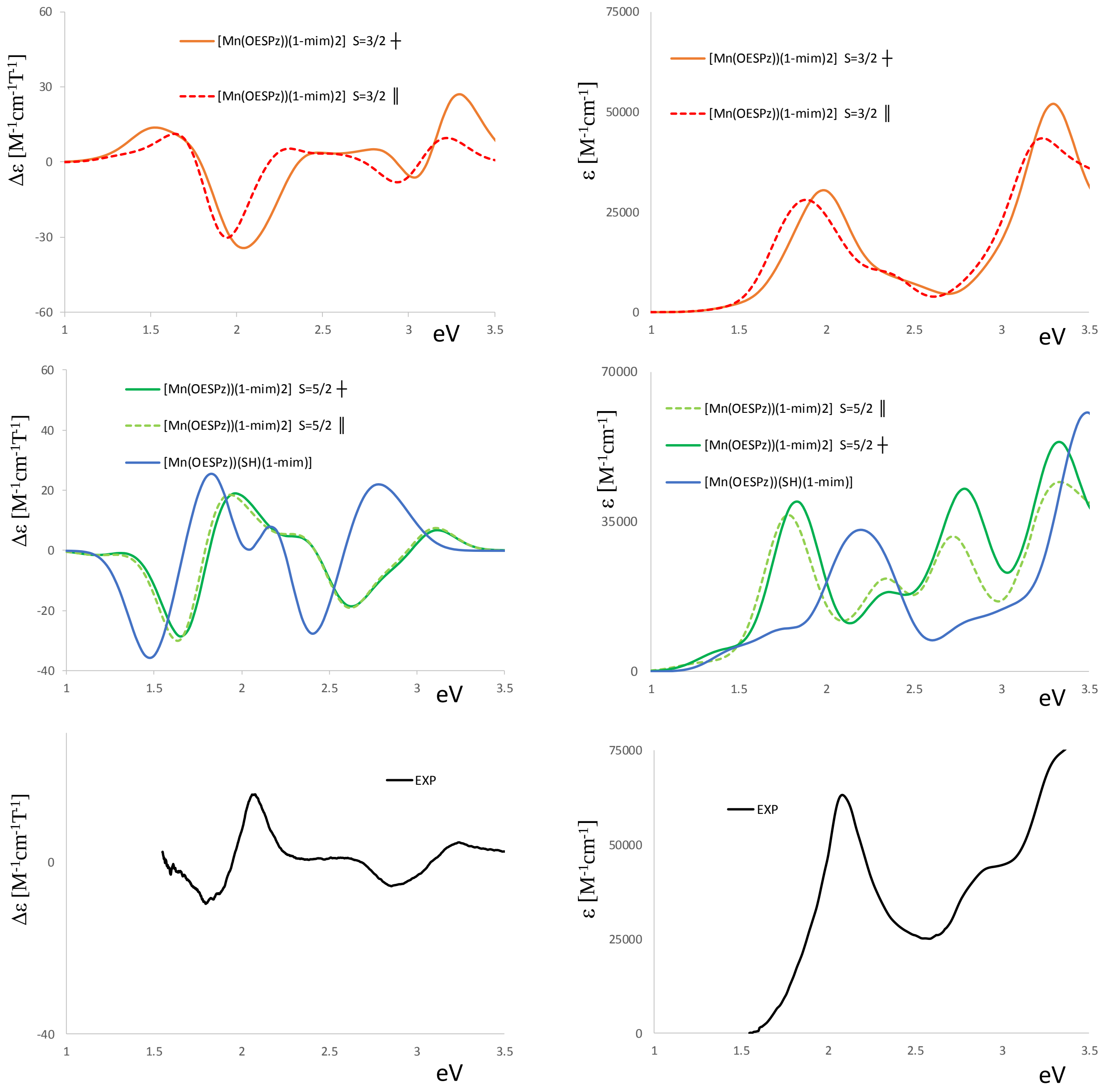

Figure S8. Comparison of the calculated MCD (*left*) and UV-vis (*right*) spectra of [Mn(OESPz)(SH)(1-mim)], [Mn(OESPz)(1-mim)$_2$] S=3/2, and [Mn(oespz)(1-mim)$_2$] S=5/2 complexes (*left*) and the corresponding experimental spectrum (*right*). For the case of [Mn(OESPz)(1-mim)$_2$] two possible orientations of the 1-mim groups (parallel and perpendicular) have been considered.

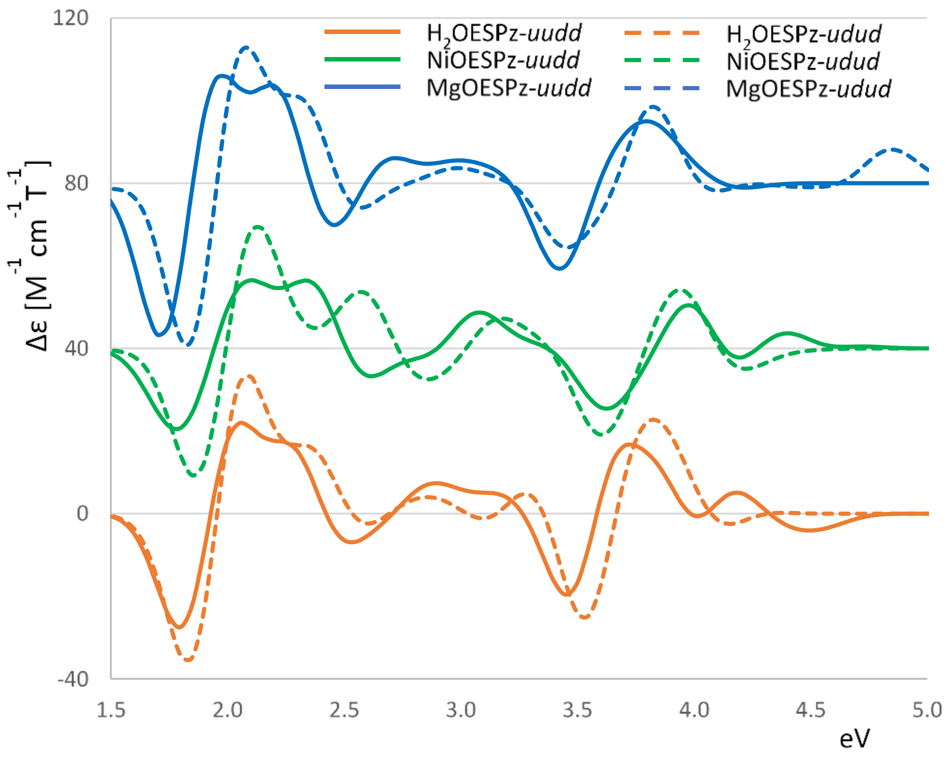
120
80
40
0
-40
Δε [M-1 cm-1 T-1]
1.5
2.0
2.5
3.0
3.5
4.0
4.5
5.0
eV
H2OESPz-uudd
NiOESPz-uudd
MgOESPz-uudd
H2OESPz-udud
NiOESPz-udud
MgOESPz-udud

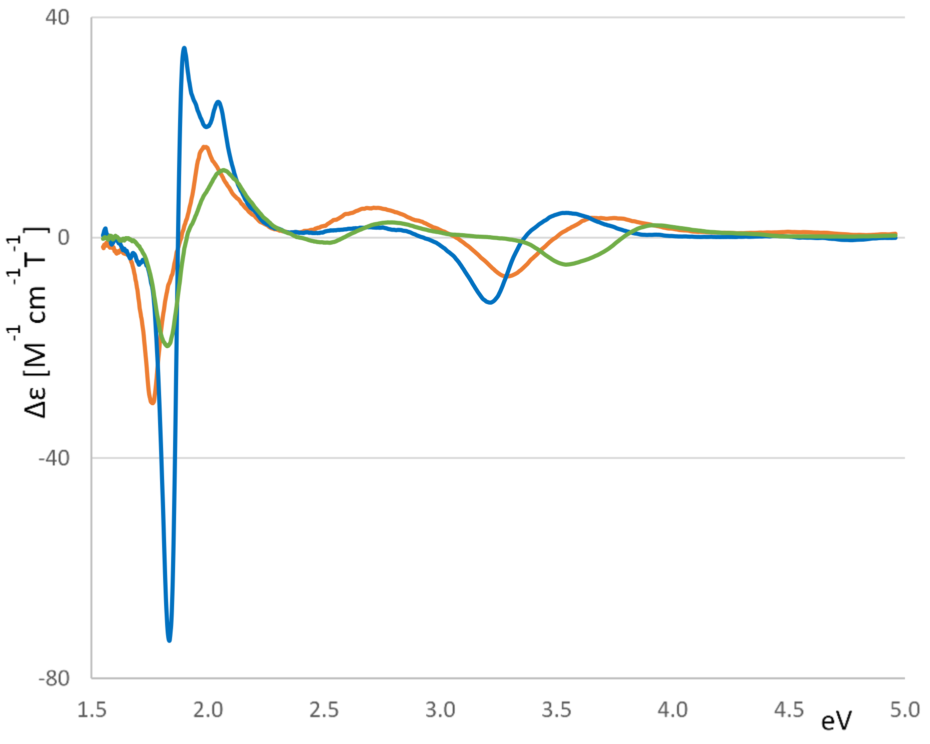
40
0
-40
-80
Δε [M-1 cm-1 T-1]
1.5
2.0
2.5
3.0
3.5
4.0
4.5
5.0
eV

Figure S9. Comparison of the calculated MCD spectra of ZnOESPz, MgOESPz and H_2OESPZ complexes (*top*) and their corresponding experimental MCD spectrum (*bottom*).
